# Protein-mediated stabilization and nicking of the nontemplate DNA strand dramatically affect R-loop formation in vitro

**DOI:** 10.1073/pnas.2509309122

**Published:** 2025-09-18

**Authors:** Ethan Holleman, Thomas E. Catley, Tadas Sereiva, Stella R. Hartono, Alice L. B. Pyne, Frédéric Chédin

**Affiliations:** ^a^Department of Molecular and Cellular Biology, University of California, Davis, CA 95616; ^b^Department of Chemical, Materials and Biological Engineering, University of Sheffield, Sheffield S1 3JD, United Kingdom

**Keywords:** R-loop formation, single-stranded DNA binding proteins, DNA nicks, atomic force microscopy

## Abstract

R-loops are an important class of non-B DNA structures that form co-transcriptionally. Using in vitro transcription and unbiased quantitative sequencing readouts, we show that the addition of single-strand DNA binding proteins co-transcriptionally can drive a 3- to 5-fold increase of R-loop frequency without significant changes to R-loop distribution. We propose that this is caused by stabilizing and preventing the collapse of short nascent R-loops. This suggests that R-loop formation is highly dynamic and highlights single strand binding proteins as players in cellular R-loop regulation. We further show that nontemplate strand DNA nicks are powerful initiators of R-loop formation, increasing R-loop frequencies by up to two orders of magnitude. Atomic force microscopy revealed that the nontemplate strand in nick-initiated structures is often flayed away from the RNA:DNA hybrid and engaged in self-pairing, creating unique forked R-loop features. DNA nicks, one of the most frequent DNA lesions in cells, are therefore potential hotspots for opportunistic R-loop initiation and may cause the formation of a distinct class of R-loops. Overall, this work highlights the importance of the displaced single-strand on R-loop initiation and dynamics.

## Significance

R-loops are three-stranded DNA:RNA hybrid structures that form during transcription and play critical roles in both gene regulation and genome stability. This study reveals that single-stranded DNA-binding protein increases R-loop frequency by stabilizing nascent R-loops, revealing a role for this protein in R-loop dynamics. Additionally, single strand DNA breaks, common cellular lesions, are identified as potent initiators of R-loops, increasing their rate of formation by up to 100-fold. Using atomic force microscopy, the study uncovers unique forked structures in nick-initiated R-loops, representing a distinct class of secondary R-loop structures. These findings provide insights into how cellular factors and DNA damage influence R-loop formation, with implications for understanding genome instability in health and disease.

R-loops are three-stranded nucleic acid structures that form during transcription upon reannealing of the nascent RNA to the template DNA strand. These structures have been described in bacteria, yeasts, plants, metazoans, and mammals (1) where they form prevalently over transcribed regions. Under physiological conditions, R-loops are well tolerated and associated with a number of adaptive processes including transcription termination (2), (3) chromatin patterning (4), class switch recombination (5), and the regulation of gene expression (6). At the same time, harmful R-loops have been implicated as players in a variety of maladaptive cellular processes, in particular as threats to genomic stability (6–8), and a number of human diseases have been linked to deregulated R-loop metabolism (9). Despite the growing recognition of the complex roles played by R-loops, significant gaps remain in our understanding of the fundamental principles guiding R-loops initiation, stability, and decay. Biochemical reconstitution of R-loop formation using in vitro transcription studies combined with mathematical modeling have identified DNA sequence and DNA topology as key factors regulating R-loop formation. R-loops are favored over GC-rich and GC-skewed regions owing to the relative energetic favorability of the resulting RNA:DNA hybrids over the corresponding DNA duplex (10). R-loops are also favored on negatively supercoiled DNA templates (11–13) owing to their ability to absorb negative superhelicity locally and to relax the surrounding DNA fiber to a lower energetic state (12). Recent mathematical modeling of R-loop energetics at equilibrium revealed that R-loop formation becomes favorable when the combined energetic return due to DNA sequence and DNA topology is such that the energetic barrier caused by the formation of Y-junctions at the start and end of R-loops can be overcome (12). It is likely, however, that additional factors beyond DNA sequence and topology play important, yet-to-be understood, roles in driving R-loop formation or stability.

The impact of the nontemplate DNA strand on R-loop formation, dynamics, and stability, has not been fully considered. This strand is in competition with the nascent RNA for reannealing to the template DNA strand (14, 15). DNA reannealing will affect the ability of an R-loop to initiate by preventing the formation of the RNA:DNA hybrid, or, if reannealing occurs after R-loop initiation, it could cause the collapse of nascent R-loops and the displacement of the RNA. This competition model predicts that stabilization of the looped-out nontemplate strand may reduce R-loop collapse. Several studies have suggested that G quadruplex (G4) formation on the G-rich displaced strand stabilizes R-loops due to the fact that that strand is now self-paired and additional energy is required to melt it back to ssDNA before it could reanneal (16) (17). Binding of single-stranded DNA binding proteins on the displaced strand may play a similar role, although this has not been formally assessed in in vitro transcription studies. Replication Protein A (RPA) was reported to catalyze R-loop formation in vitro (18) and to bind to R-loops in vivo, facilitating the recruitment of the R-loop resolving enzyme Ribonuclease H1 (RNase H1) (19). However, its role during co-transcriptional R-loop formation has not been measured. It has also been reported that single-stranded DNA breaks (nicks) in the nontemplate strand can stimulate R-loop formation (20). This effect was attributed to an increased ability of the nontemplate strand downstream of the nick to dissociate from the template DNA strand, perhaps through local breathing, facilitating RNA invasion and RNA:DNA hybrid formation. However, the positions, lengths, or characteristics of the R-loops associated with nicks were not determined, and the impact of nicks on R-loop formation frequency was not quantitatively assessed.

Here, we used in vitro transcription assays on defined plasmid substrates followed by single-molecule R-loop footprinting [SMRF-seq (13)] to quantitatively measure the impact of single-stranded DNA binding proteins and nontemplate DNA nicks on R-loop formation. This work reveals that each of these factors exerts a profound effect on the R-loop landscape that is often equally or more powerful than the well-documented effect of DNA sequence and DNA topology.

## Results

### Single Strand Binding Proteins Stabilize Nascent R-Loop Formation.

In an R-loop, the RNA strand of the RNA:DNA hybrid and the nontemplate DNA strand compete for binding to the template DNA strand. If the DNA strand prevails, the R-loop will not form, or it will collapse post-initiation. Here, we tested the hypothesis that single-strand DNA binding proteins may favor R-loop formation by stabilizing the nontemplate DNA strand. To test this, we used in vitro transcription (IVT) reactions with the T7 RNA polymerase on various R-loop prone templates, focusing first on the effect of the *Escherichia coli* single-stranded DNA binding protein (SSB). Under co-transcriptional conditions (co-txn), SSB was added to the reaction prior to transcription so that SSB may interact with R-loops from the moment they initiate. As a control, we first performed IVT, and SSB was only added after the transcription was terminated, such that SSB may only interact with R-loops that exist at the end of the reaction (post-txn).

**Fig. 1.**
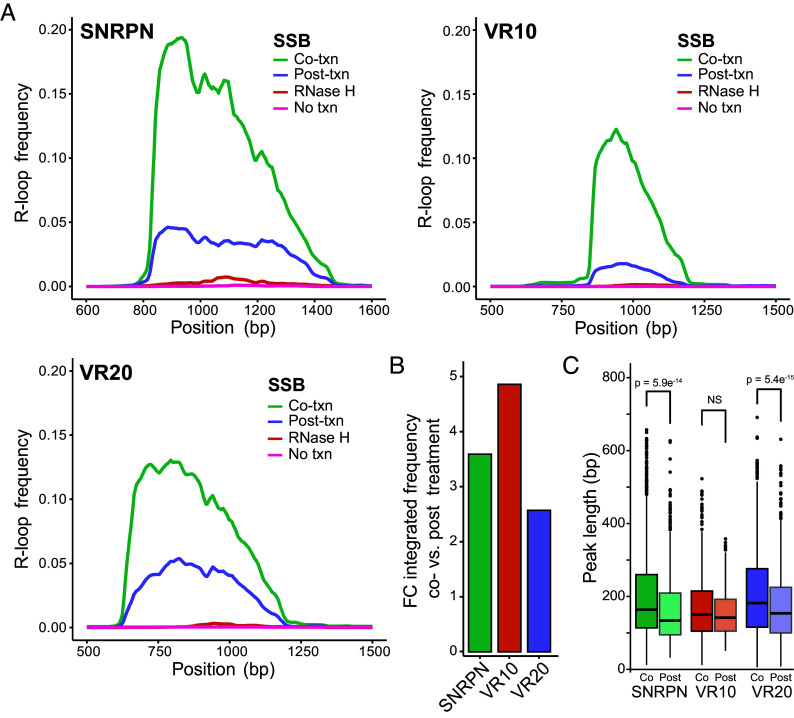
(*A*) R-loop frequency plots calculated from SMRF-seq data for linearized SNRPN, VR10, and VR20 containing plasmids. (*B*) Boxplot of R-loop peak length measured via SMRF-seq comparing co- and post-transcriptional SSB treatments for the four previously mentioned linearized plasmid substrates. *P* values calculated using Student’s *t* test. (*C*) Boxplot of fold change in R-loop frequency by base pair between co- and post-SSB treatment of the VR containing region from the VR-10 and VR-20 plasmids.

Co-transcriptional SSB addition led to a slight but discernible RNase H-sensitive mobility shift on agarose gels that was not seen upon addition of SSB post-transcription (*SI Appendix*, Fig. S1*A*). To quantitatively measure R-loop frequencies and positions in an unbiased manner, we performed SMRF-seq after IVT. Three different linear plasmid constructs, each with a different background propensity for R-loop formation based on DNA sequence, were used. Overall, adding SSB co-transcriptionally led to a remarkable 3- to 5-fold increase in R-loop frequencies compared to SSB addition post-transcription (Fig. 1 *A* and *B*). As expected, R-loop peaks measured by SMRF-seq were highly strand specific and located to the nontemplate strand. R-loop footprints were also highly sensitive to RNase H pretreatment prior to SMRF-seq (*SI Appendix*, Fig. S1*B*). Interestingly, R-loop locations did not significantly change upon SSB addition (Fig. 1*A* and *SI Appendix*, Fig. S1*C*). These observations are most consistent with the notion that SSB binds to the exposed nontemplate strand of an R-loop once it becomes available after an R-loop has initiated. SSB binding is proposed to stabilize that strand and lower the chances of early R-loop dissolution by spontaneous DNA reannealing. The strong effect of SSB on R-loop frequencies indicates that, in the absence of SSB, the large majority of R-loops collapse before they can be measured. Interestingly, it should be noted that all samples here were subjected to deproteinization prior to SMRF-seq. This suggests that the increase in R-loops observed upon co-transcriptional SSB addition was derived from a population of R-loops that were prone to early collapse without SSB, but did not require SSB for their long-term stability once fully formed. Thus, the effect of SSB is most likely felt early during R-loop formation through the stabilization of short nascent R-loops.

We measured the lengths of R-loops formed under co- and post-transcriptional conditions to determine whether SSB also allows the growth of R-loops during R-loop elongation. Median R-loop lengths under post-transcriptional SSB addition ranged from approximately 120 to 150 bp, consistent with prior findings (12). A modest increase in length was observed for two out of the three tested constructs (Fig. 1*C*). This indicates that stabilization of the nontemplate strand may allow for slightly more R-loop extension compared to an unstabilized nontemplate strand. The effect size, however, was small compared to the increase in overall R-loop frequency suggesting that nontemplate strand stabilization plays a comparatively minor role in R-loop elongation.

### Transcription Through Nicks Increases R-Loop Formation Regardless of Sequence or Distance from Promoter.

Nicks may allow the displaced DNA strand to locally dissociate from the DNA template strand, thereby favoring the reannealing of the nascent RNA to initiate an R-loop (20). Alternatively, or in addition, we propose that R-loops that initiate at, or in the immediate vicinity of nicks would not have to “pay” the energetic cost imposed by the formation of a Y junction. An R-loop initiated downstream of a nick indeed does not have to contend with a start junction due to the interruption of the phosphate backbone. If correct, this hypothesis predicts that R-loop formation following a nick should be less constrained by the favorability of the downstream DNA sequence, due to the lower requirement for energy return from RNA:DNA hybrid formation.

**Fig. 2.**
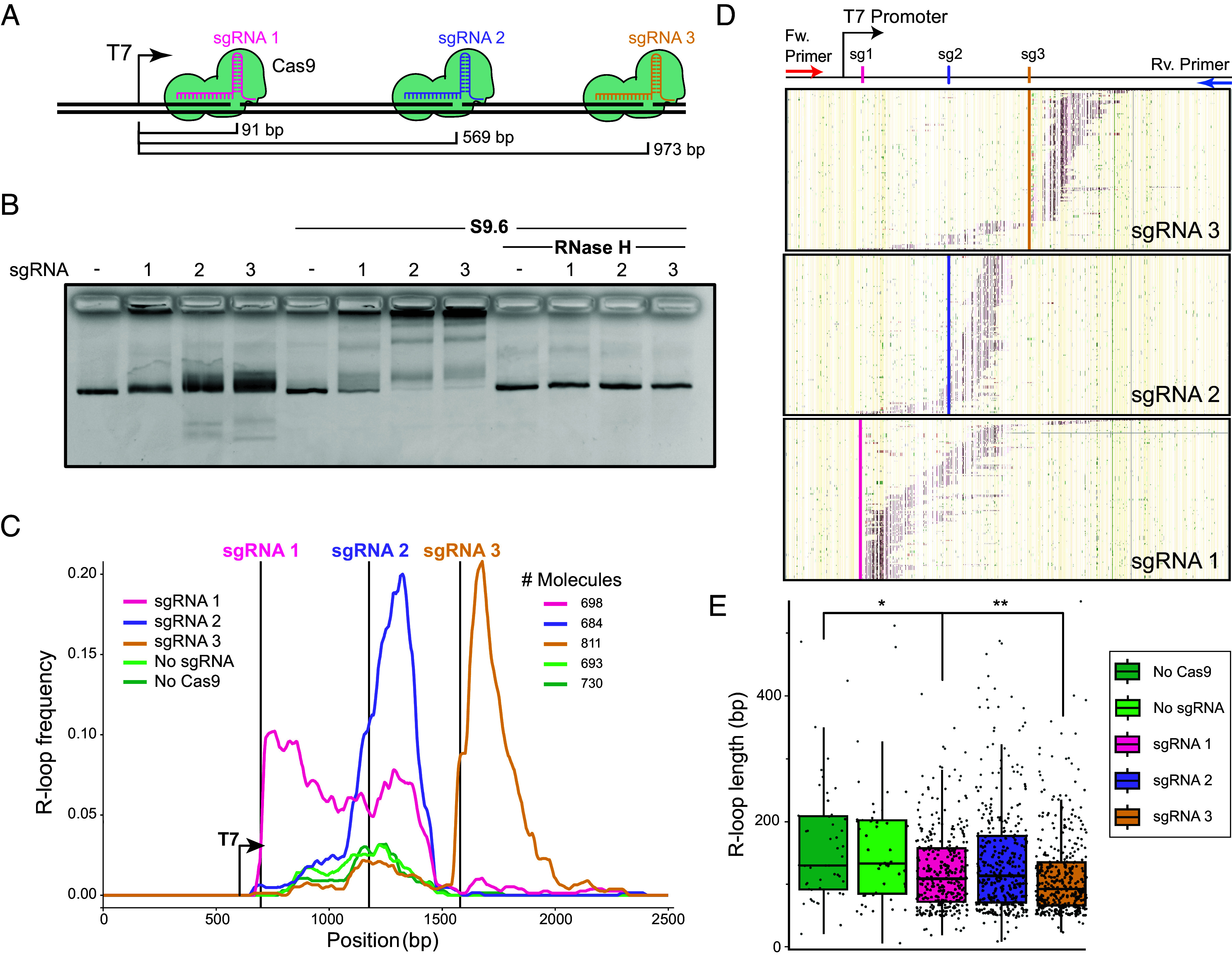
(*A*) Diagram showing target sites of the three sgRNAs used to induce Cas9 strand-specific DNA nicks relative to the T7 transcription start site. (*B*) Agarose gel electrophoresis of in vitro transcription reaction on nicked and unnicked linearized plasmids. S9.6 was added post-transcription as indicated to cause supershifting, while RNase H treatment validates RNA:DNA hybrid formation. (*C*) Population-average frequency plot of SMRF-seq signal from in vitro transcription reactions on the nontemplate DNA strand. Locations of Cas9 nicks are shown as vertical lines; the position of the T7 promoter is indicated. The number of independent DNA molecules analyzed by SMRF-seq is indicated. (*D*) A representative sampling of individual SMRF-seq footprints collected for each nicked plasmid is displayed. 200 independent R-loop-carrying molecules are displayed vertically stacked. The position of cytosines is shown by thin yellow tick marks. Red tick marks indicate cytosines that were single-stranded and part of an R-loop footprint. Green tick marks indicate cytosines that showed single-stranded character but not considered to be part of a footprint. Nick positions are indicated. (*E*) Boxplot of individual R-loop lengths measured in base pairs for nicked and unnicked conditions.

To test this hypothesis, we utilized a CRISPR-Cas9 nickase system (21, 22) and three single guide RNAs (sgRNAs) (23) to introduce DNA nicks in the nontemplate strand of an R-loop-prone plasmid carrying the human *SNRPN* gene (12, 24). These sgRNA molecules, termed sgRNA 1, 2, and 3 directed strand-specific Cas9-mediated nicks 91, 569, and 973 bp downstream of the T7 transcription start site (TSS), respectively (Fig. 2*A*). Analysis of the local average R-loop energy for the first 100 bp downstream of each nick, showed that the sequence following sgRNA 2 was by far the most favorable (*SI Appendix*, Fig. S2*A*), corresponding to a well-known GC-skewed R-loop formation hotspot (12). The region following sgRNA 1 was moderately favorable, while the region downstream of sgRNA3 was the least favorable and the furthest downstream from the promoter.

The induction of Cas9-mediated nicks in supercoiled plasmid templates was observed via a characteristic loss of mobility during agarose gel electrophoresis (*SI Appendix*, Fig. S2*B*). Only reactions showing at least 95% efficiency were utilized in downstream experiments. The location of the nicks was verified using Sanger sequencing by a tell-tale early termination of the sequencing reaction at the expected nick site (*SI Appendix*, Fig. S2*C*). Having validated a robust Cas9-based nick induction methodology, we next qualitatively determined the impact of nicks on R-loop formation in bulk by performing in vitro transcription reactions utilizing T7 RNA polymerase followed by electrophoretic mobility shift assays. All substrates were deproteinized and linearized prior to transcription to remove DNA-bound Cas9 complexes and any effect of DNA topology, respectively. R-loop formation was inferred by a characteristic upward gel shifting. Treatment with sgRNA 1, 2, and 3 resulted in increased upward shifting that was greatly accentuated by incubation with the anti-RNA:DNA hybrid S9.6 antibody (25) prior to electrophoresis (Fig. 2*B*). We confirmed that gel shifting reflected R-loop-mediated phenomena by treating transcribed substrates with purified RNase H prior to gel electrophoresis. This treatment completely eliminated S9.6 binding. Thus, transcription of nicked substrates causes a strong increase in R-loop formation, extending prior results (20). Interestingly, all three nicked substrates responded strongly to nicks when transcribed, suggesting that the distance from the TSS to the nick, and the local DNA sequence, do not qualitatively influence the impact of a nick on R-loop formation.

### SMRF-Seq Reveals R-Loop Induction at or in the Immediate Vicinity of Nicks.

To provide quantitative and spatial insights into the effect of nontemplate strand nicks on R-loop formation, we subjected in vitro transcription reaction products to a modified SMRF-seq protocol which includes a post–bisulfite treatment nick repair step to allow for successful PCR amplification of nicked templates (*SI Appendix*, Fig. S2*D*). Importantly, SMRF-seq does not involve any enrichment for R-loop-carrying molecules and instead samples all plasmids in the reaction, providing an unbiased, quantitative, high-resolution, and strand-specific measurement of R-loop formation on single DNA molecules at ultradeep coverage.

Analysis of population-average R-loop frequency curves showed that transcription through unnicked, linear templates resulted in modest R-loop formation clustering around the most favorable, GC-skewed, portion of the transcribed region (Fig. 2*C*), as described (12). This R-loop prone region maps to the area targeted by sgRNA 2. In vitro transcription after treatment with Cas9 alone in the absence of sgRNA did not result in any significant change in R-loop frequency, as expected. In stark contrast, the bulk R-loop frequency was dramatically increased immediately at, and downstream of, the site of Cas9-induced nicks (Fig. 2*C*). For sgRNA 2 and 3, a sharp peak of R-loop formation was observed over a ~400 bp region downstream of the nicks. For sgRNA 1, increased R-loops were induced over a wider region ~800 bp downstream of the nick. RNase H treatment eliminated nearly all SMRF-seq signal confirming that it originated from bona fide R-loops (*SI Appendix*, Fig. S2*E*). Additionally, the frequency increases observed here were highly strand-specific, with no effect observed on the template DNA strand (*SI Appendix*, Fig. S2*F*), as expected from R-loops.

Analysis of individual R-loop-containing DNA molecules recovered from SMRF-seq showed that in 98.8% of cases, these molecules only carried a single R-loop, consistent with prior findings (12). In addition, SMRF-seq confirmed the presence of clear R-loop footprints downstream of the nick sites in many independent molecules (Fig. 2*D*). We determined that the structures formed downstream of the sgRNA target sites resulted from transcription events originating at the T7 promoter, since adventitious loading of the T7 RNAP at nicks did not result in significant transcription or R-loops (*SI Appendix*, Fig. S2*G*). R-loop lengths were measured from individual R-loop start and stops. For unnicked samples or Cas9-only treated samples, median R-loop lengths were 173 bp. Nick-induced R-loops were slightly smaller (Fig. 2*E*), with the most significant difference observed for R-loops induced by sgRNA 3, and to a smaller extent, sgRNA 1. As mentioned, DNA sequence energetics for the sgRNA 3 nick-downstream region was the least favorable for R-loop formation, possibly accounting for the reduced R-loop size.

### DNA Nicks Stimulate R-Loop Formation by One to Two Orders of Magnitude, Regardless of Nicking Methodology.

**Fig. 3.**
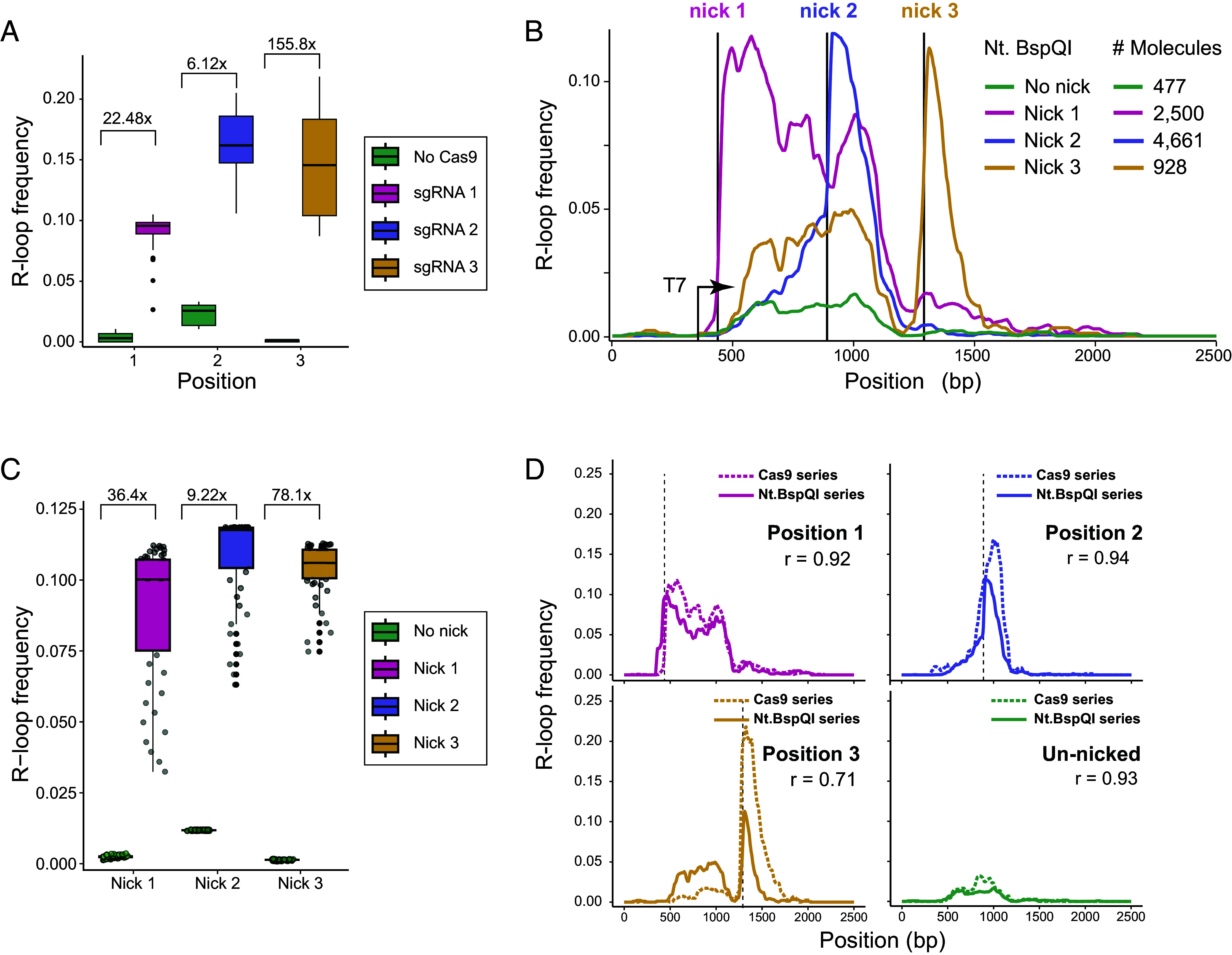
(*A*) Box plot of R-loop frequencies from SMRF-seq measured over the 50 bp region immediately following the site of Cas9-induced nicks in nicked vs. unnicked samples. The fold-change between unnicked and nicked samples is indicated above paired samples. (*B*) R-loop frequency plot from SMRF-seq data for linearized plasmids with or without Nt.BspQI-induced nicks. Horizontal lines indicate the location of Nt.BspQI recognition sites. The number of independent molecules sampled is indicated at *Right*. (*C*) As in panel *A*, except data are for Nt.BspQI-induced nicked and unnicked samples. (*D*) Comparison between R-loop frequency plots calculated from SMRF-seq signal between analogous Nt.BspQI and Cas9 nicked and unnicked samples. The plots indicate the Pearson correlation coefficient between the two curves.

We measured R-loop frequencies over a 50 bp window immediately downstream of each nick in both nicked samples and unnicked controls. R-loop frequency increased a stunning 155-fold for the sgRNA 3 site, while sgRNA 1 and 2 led to 22-fold and sixfold increases, respectively (Fig. 3*A*). The main reason for this disparity in fold-change was due to the differences in background R-loop favorability in unnicked samples. R-loop frequencies in nicked samples were relatively consistent, ranging from 9 to 16% between sgRNAs (Fig. 3*A*). This suggests that R-loop induction at nicks is similar regardless of the distance to the TSS, contrary to a previous report (20). This implies that the length of the trailing RNA transcript when the polymerase reaches the nick does not have an appreciable effect on the nick-induced R-loop frequency. It also implies that R-loop induction following a nick can be extraordinarily effective, even when the downstream DNA sequence is not intrinsically favorable to R-loops, as observed for sgRNA 3.

To verify that the increase in R-loop frequency at nicks was not Cas9-specific, we engineered the *SNRPN* region to contain an Nt.BspQI recognition site either 126, 578, or 978 bp downstream of the T7 promoter. Treatment with the Nt.BspQI nicking endonuclease allowed us to generate site-specific nicks at high efficiency in positions immediately adjacent to that of the Cas9-induced nicks and independent of any sgRNA Nt.BspQI-induced nicks greatly increased R-loop formation compared to unnicked controls in a manner highly similar to that observed for Cas9-induced nicks (Fig. 3*B*). The R-loop frequencies downstream of each nick site were similar for all three positions and the fold changes ranged from 9-fold for Nick 2, to 36-fold for Nick 1 and 78-fold for Nick 3 (Fig. 3*C*). As observed previously, nick-induced R-loops were highly specific to the nontemplate strand (*SI Appendix*, Fig. S3*A*), were not apparent in nicked but untranscribed samples (*SI Appendix*, Fig. S3*B*) and were abrogated by RNase H treatment (*SI Appendix*, Fig. S3*C*). Direct comparison of R-loop frequency curves confirmed a high correspondence between Nt.BspQI-induced and Cas9 nickase-induced samples (Fig. 3*D*), establishing that nicks are powerful R-loop initiators regardless of nicking strategy or distance from the TSS.

The ability of nicks to stimulate R-loop formation regardless of the downstream sequence is consistent with the idea that the energetic cost of sustaining a Y-junction is the major bottleneck to structure formation (12). Our dataset provides an opportunity to adapt our R-loop equilibrium energy model, R-looper, to simulate nick-induced R-loop formation and to empirically estimate the junction energy. We found that the current R-loop junction energy values, based on a simple strand separation model (26), likely underestimate the energetic cost. The best fit between predicted and experimental data was observed at a junctional energy (a) value of 17.9 kcal/mol, representing a 1.79x fold increase compared to the previously utilized (a) value (*SI Appendix*). Importantly, the modeling confirmed that simply relieving the junction energy at one base-pair can result in a dramatic shifting of R-loop distribution to that position, in effect creating a major R-loop hotspot (*SI Appendix*).

Given the distinct mechanisms by which nicks and SSB stimulate R-loop formation we aimed to determine how these factors interact. To do so, we used a template carrying a Nt.BspQI-induced nick at site 1, and performed IVT using SSB added co-transcriptionally or post-transcription. Co-transcriptional SSB addition led to increased R-loop formation compared to adding SSB post-transcription (*SI Appendix*, Fig. S3*D*). The distribution of R-loops, however, did not change as both curves closely matched each other, and showed clear R-loop induction at the site of the nick. As expected, R-loop formation was dependent on transcription, sensitive to RNase H pretreatment (*SI Appendix*, Fig. S3*D*), and unique to the nontemplate strand (*SI Appendix*, Fig. S3 *D* and *E*). Co-transcriptional SSB addition further increased R-loop formation downstream of Nick 1 by 1.7-fold compared to nicking alone (*SI Appendix*, Fig. S3*F*). This indicates that a proportion of nascent R-loops initiated at a nick are also prone to collapse and become stabilized by SSB. The fold change, however, was smaller than that observed for unnicked plasmids (1.7-fold versus 3- to 5-fold) indicating that R-loops initiating at nicks may be less sensitive to collapse. This is expected given that nick-induced R-loops should be less susceptible to the energetic burden caused by the initiating Y-junction.

### Direct Visualization of R-Loops Formed at Nicks Reveal Unique and Complex “Forked” Secondary Structures.

**Fig. 4.**
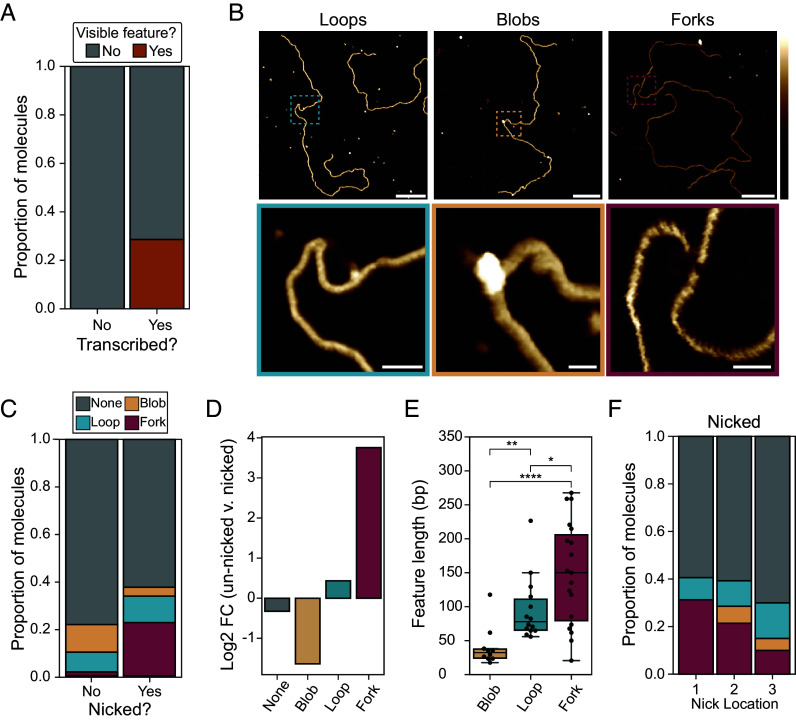
(*A*) Barplot showing the proportion of molecules with any visible feature type observed using AFM on transcribed and untranscribed molecules. (*B*) Representative AFM images of three observed structure classes; forks, loops, and blobs. (Scale bars on *Upper* images represent 100 nm.) *Lower* image (Scale bars are equal to 10 nm.) Height scale = −1 to 3 nm. (*C*) Barplot showing the proportion of each feature type (or lack of feature) observed via AFM on transcribed nicked and unnicked samples. (*D*) Barplot showing fold change between proportion of each feature type between unnicked and nicked transcribed molecules observed using AFM. (*E*) Boxplot showing distribution of feature lengths by type in base pairs. (*F*) Boxplot showing proportion of feature types observed for each nick location.

Atomic force microscopy (AFM) of in vitro generated R-loops has been used previously to characterize the resulting structures (27). Given the significant increases in R-loop frequency observed at nicks, we wanted to determine whether these structures resembled those formed in the absence of nicks. A total of 287 molecules were imaged, including 144 Nt.BspQI-nicked and 143 unnicked molecules. The median contour length of all molecules was estimated to be 3473 bp (1181 nm), a 2.3% deviation from the expected plasmid length (*SI Appendix*, Fig. S4*A*). As expected, untranscribed molecules, whether nicked or unnicked, did not carry any structural feature deviating from that expected from B-DNA. This confirms that nicks in the absence of transcription did not induce secondary structures. Structural features deviating from B-DNA were observed on approximately 30% of transcribed molecules (Fig. 4*A*). Within this subset, three distinct classes of features were observed and categorized as loops, blobs, and forks (Fig. 4*B* and *SI Appendix*, Fig. S4*B*). Only one feature was detected per molecule. Loops and blobs have previously been reported as bona fide R-loop features (27); forks, however, are unique transcription-induced features. Forks are characterized by the appearance of an unexpected segment of double-stranded nucleic acid jutting from the main molecule and seemingly connected by what often appears to be ssDNA (Fig. 4*B*). Some fork objects also displayed various degrees of complexity, with the appearance of branched structures (*SI Appendix*, Fig. S4*B*).

Transcription through unnicked plasmids produced a large number of loop and blob features, consistent with prior observations (Fig. 4*C*) (27). 21.6% (13 molecules out of 60) of all transcribed unnicked molecules carried a visible R-loop feature; only one molecule carried what appeared to be a fork. Transcription through nicked samples resulted in a significant increase in the overall proportion of molecules carrying a feature: 33.8% (27 molecules out of 80) of transcribed nicked molecules carried a visible non-B DNA feature. Thus, nicks induced a 1.74-fold increase in the frequency of non-B DNA features detected by AFM. In sharp contrast to unnicked samples, the majority of molecules resulting from transcription through nicked substrates carried fork features (Fig. 4 *C* and *D*). Overall, there was a dramatic ~13-fold increase in the number of fork features between nicked and unnicked transcribed samples, while the proportion of blobs decreased nearly threefold (Fig. 4*D*). Features in nicked samples were significantly longer than those observed in unnicked samples; this increase was primarily driven by the fact that fork objects are larger than blobs or loops (Fig. 4*E*). Forks had lengths averaging 149 bp (± 76 bp), while loops and blobs had average lengths of 96 bp (± 46 bp) and 41 bp (± 30 bp), respectively. The relative proportion of fork objects varied depending on the nick position, with nick position 1 showing the most forks, and nick position 3 the least (Fig. 4*F*). Overall, fork objects represent a distinct nick-induced, transcription-dependent, class of R-loop structures.

### Fork Structures Most Likely Result from Nontemplate Strand “Peeling” and Self-Folding During R-Loop Formation.

**Fig. 5.**
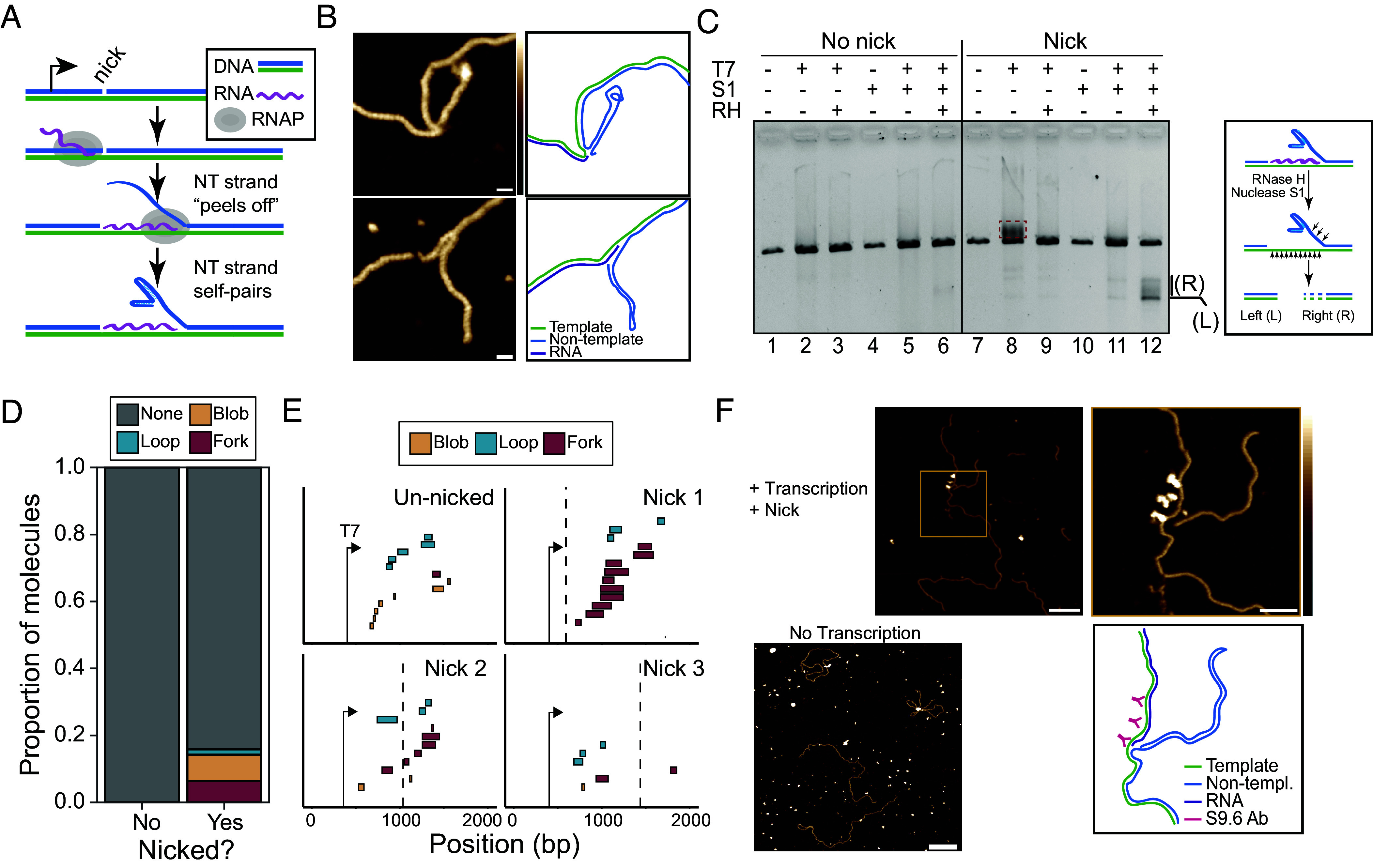
(*A*) Cartoon diagram of model of an R-loop associated fork structure at the site of a nontemplate strand nick. (*B*) AFM images and corresponding strand tracing diagram models of two fork structures observed on transcribed and nicked substrates. (Scale bar, 10 nm.) Height scale = −1 to 3 nm. (*C*) Agarose gel electrophoresis of an in vitro transcription reaction on linearized nicked and unnicked SNRPN containing plasmid. Pluses or minuses above lanes indicate the addition or absence of a particular enzyme in the reaction. The symbols T7, S1, and RH correspond to T7 RNA polymerase, S1 nuclease and RNase H1 (*E. coli*) respectively. The *Inset* at right depicts our model for the molecular events upon combined RNase H and Nuclease S1 treatment. (*D*) Barplot showing the proportion of feature types observed via AFM in nicked and unnicked transcribed and RNase H1 treated molecules. (*E*) Footprint plot showing the locations of features with respect to each nick location and the T7 promoter seq. The expected nick site is indicated by a vertical dashed line. (*F*) AFM image and strand tracing cartoon model of a nicked and transcribed plasmid treated with S9.6 antibody. (Scale bar on *Left* image = 100 nm.) (Scale bar on *Right* image = 40 nm.) Height scale = −1 to 3 nm. An AFM image on untranscribed samples is shown at *Bottom Left* with abundant S9.6 presence away from the DNA molecules. (Scale bar, 40 nm.)

We hypothesized that the unique and unexpected fork objects could be explained by the nontemplate strand peeling from the template downstream of the nick, as it is not covalently attached to the upstream-of-nick nontemplate strand. We further hypothesized that this strand may fold back onto itself forming a variety of possible hairpin-like structures that would result in a dsDNA character when observed via AFM (Fig. 5*A*). Further examples of fork structures with corresponding inferred strand positions and orientations are shown in Fig. 5*B*. To test this hypothesis in bulk, we transcribed nicked substrates and treated them simultaneously by RNase H and low levels of nuclease S1. We reasoned that if the RNA portion of the RNA:DNA hybrid was rapidly removed by RNase H, the fork structure would likely impose a slight kinetic delay before the displaced strand could reanneal due to the additional stability afforded by its secondary structure. RNase H digestion would therefore leave a region of exposed ssDNA on the template strand with the approximate size of the RNA:DNA hybrid (Fig. 5*C* and *SI Appendix*, Fig. S5*A*). This ssDNA should be hypersensitive to S1 digestion. The self-paired fork structure itself should be sensitive to S1 cleavage in places where ssDNA is exposed. This is expected to lead to the breakage of the linear DNA, with two products expected. One corresponds to a left fragment of defined size running from the end of the linear molecule to the nick. The second right fragment should be of variable size depending on the position and length of the RNA:DNA hybrid and the geometry of the self-paired fork (Fig. 5*C* and *SI Appendix*, Fig. S5*A*). These digestion products should be apparent by agarose gel electrophoresis given a high enough concentration of forks were present. Regular R-loops formed outside of a nick or on unnicked templates are expected to resolve back to dsDNA immediately after the RNA strand is digested by RNase H.

As expected, we found minimal S1 nuclease digestion products in the unnicked transcribed and RNase H-treated sample (Fig. 5*C*, lane 6), consistent with rapid R-loop resolution back to dsDNA upon RNase H treatment. Transcription of the Nt.BspQI-nicked substrate led to significant upward smearing compared to an unnicked substrate (compare lanes 2 and 8), consistent with increased R-loop formation. S1 treatment of transcribed nicked molecules led to the disappearance of the smear (compare lanes 11 and 8), consistent with it being due to secondary structure formation on the displaced strand, as proposed (27). Co-treatment with S1 nuclease and RNase H led to the clear appearance of new digestion products compared to S1 treatment alone (compare lanes 12 and 11), indicating that the new ssDNA substrates for S1 nuclease were generated by RNase H activity, as hypothesized. The products appeared as a lower band and an upper smear (Fig. 5*C*). The lower product closely corresponded in size to the distance expected between the BsaI site used to linearize the plasmids and the Nt.BspQI target site. The defined character and size of this band are entirely consistent with this product corresponding to the expected left product. The upper smear, in turn, is consistent with the products expected from S1 nuclease cleavage of forks of variable structures and of RNase H-exposed ssDNA of variable position and lengths originating from the right side of plasmid substrates. There was minimal visible S1 digestion on nicked untranscribed samples (lane 10), indicating that the digestion products were both transcription- and RNase H-dependent, as expected.

Our hypothesis for the origin of fork objects makes three further predictions. First, RNase H treatment of fork objects may not result in a full return to dsDNA due to the intrinsic stability of the self-paired nontemplate strand. Second, fork objects should appear after nicks. Third, the ability of a given region to form fork objects should depend on the ability of the region to undergo self-folding. To test the first prediction, we applied AFM on RNase H-treated nicked and unnicked transcribed plasmids. As expected, no visible structures were observed in any of the RNase H-treated unnicked samples (61 total molecules), consistent with complete resolution to B-form dsDNA (Fig. 5*D*). However, a relatively large proportion of residual structures persisted in the nicked samples post RNase H treatment. These residual structures were mostly composed of blobs and forks (Fig. 5*D*). The presence of fork objects post RNase H treatment is consistent with the notion that self-paired structures on the nontemplate strand may remain stable and resist unwinding and reannealing to the template strand. It is possible that blob objects represent collapsed forks, although this hypothesis requires additional investigation. To test the second prediction, we plotted the location of all observed structures for the three nicked and unnicked conditions after AFM imaging and careful recording of the position of each structure relative to the ends of the molecule. We found that, in general, forks tended to be located post nicks (Fig. 5*E*). For nick 1, all structures were found post nick although we note that the distance from the T7 promoter to the nick is short in this configuration. For nick 2, 5 out of 6 fork objects were located post nick. For nick 3, only one fork object was observed, and it was located prior to the nick. Quantification of these data showed that forks showed a clear bias in their position, occurring in 80% of the cases after the nick position. By contrast, blobs and loops showed no biases in their location and were nearly equally likely to occur before or after nick sites (*SI Appendix*, Fig. S5*B*). From this, we conclude that fork objects typically occur downstream of nick sites. To test the third prediction, we first investigated the self-folding potential of the *SNRPN* region in which nick positions 1 and 2 are located. Dot-plot analysis showed a large number of both direct and indirect repeats, indicating the sequence’s folding potential (*SI Appendix*, Fig. S5*C*). Additionally, mean free energy based folding predictions confirmed significant pairing capacity, with a number of hairpin structures visible and ~54% of nucleotides paired (*SI Appendix*, Fig. S5*D*). Since we noted that the proportion of fork objects was highest for Nick 1 compared to Nick 2, and lowest for Nick 3 (Fig. 4*F*), we next used secondary structure prediction algorithms to determine the self-folding probability of regions downstream of each nick. We observed that the sequence around Nick 1 had the lowest (most favorable) predicted folding energy while Nick 2 showed an intermediate prediction and Nick 3 is positioned in a relatively unfavorable region for folding (*SI Appendix*, Fig. S5*E*). Overall, these results are consistent with our hypothesis that fork structures arise by peeling and self-folding of the nontemplate strand during R-loop formation.

As a final test of the hypothesis, we wished to test for the presence of an RNA:DNA hybrid portion next to the fork object itself. Since AFM alone cannot easily differentiate RNA:DNA hybrid from dsDNA, we took advantage of the S9.6 anti-RNA:DNA hybrid antibody to mark hybrids and performed AFM imaging on nicked and unnicked substrates. As expected, we observed minimal S9.6 binding to plasmid molecules in the untranscribed nicked condition, indicating absence of RNA:DNA hybrids or R-loops (Fig. 5*F*). Clear S9.6 binding was observed on nicked and transcribed samples, often appearing as large aggregates of binding indicating R-loop formation but making detailed structural interpretation difficult (*SI Appendix*, Fig. S5*F*). We were able to observe a clear instance where S9.6 binding was located immediately adjacent to the protruding fork, but was absent from the fork itself (Fig. 5*F*). This is predicted if the fork feature itself was a dsDNA structure immediately adjacent to an RNA:DNA hybrid.

## Discussion

R-loop formation is influenced by the favorability of the underlying DNA sequence and by the level of negative DNA superhelicity available (12, 28). Our work here reveals that the nontemplate DNA strand exerts profound additional impacts on R-loop formation independently of DNA sequence or topology. Consistent with the notion that the nascent RNA competes with the nontemplate DNA strand for binding to the template DNA strand during R-loop formation (20), we show that binding of the nontemplate DNA strand by *E. coli* SSB strongly stimulated R-loop frequency by up to 5-fold. Importantly, this effect required addition of SSB co-transcriptionally such that SSB could interact with the displaced strand of R-loops as soon as it became available. Furthermore, SSB was not necessary to ensure R-loop stability after the transcription reaction was complete. We interpret this to mean that SSB stabilized short nascent R-loops that would have otherwise collapsed back to duplex DNA during early R-loop formation. This is consistent with the view that forming an R-loop involves a significant energetic cost in the form of creating at least one Y-junction at the point where the nascent RNA invades the duplex DNA. This energetic cost can be “repaid” by the formation of a more stable RNA:DNA hybrid and by the relaxation of negative supercoiling (12). However, this energetic return from RNA:DNA hybrid formation and superhelical relaxation scales with size, rendering short R-loops particularly vulnerable to early collapse. SSB can bind to as little as 35 nucleotides of ssDNA per tetramer (29), ensuring that binding to the displaced nontemplate strand can occur early. SSB binding is expected to increase as more ssDNA becomes available during R-loop elongation, resulting in the stabilization of the displaced DNA strand and in a reduced tendency for R-loop collapse. According to this model, the effect of SSB should only be manifested in the stabilization of R-loops that have already initiated and for which an ssDNA platform is available. Indeed, while SSB significantly increased R-loop frequency, it did not shift the locations of R-loops. SSB binding only had a minor effect of R-loop lengths suggesting that its main impact is on R-loop initiation. Overall, this work shows that R-loop formation is highly dynamic and that in the absence of stabilizing factors, most R-loops that initiate end up in spontaneous early collapse due to the reannealing of the nontemplate DNA strand. Our findings suggest that single-stranded DNA binding proteins may play an important role in regulating R-loop formation in cells. Conditions where SSB or RPA availability is limiting, including conditions of replication stress, genotoxic damage (30, 31), or unprocessed RNA accumulation (32), might be expected to show reduced R-loop loads due to early collapse. Likewise, it is possible that RPA availability may regulate the resolution of R-loops by Ribonuclease H1 given the proposed role of RPA in recruiting this R-loop resolvase (18).

Our work further demonstrates that nicks in the nontemplate DNA strand can increase R-loop frequencies by up to two orders of magnitude, dramatically extending previous observations (20). Our findings further demonstrate that the effect of nicks is largely independent of both the underlying DNA sequence and the distance of the nick to the transcription start site. The impact of nicks on R-loop frequencies can be most easily explained by the high energetic cost of creating a Y-junction when initiating an R-loop. If an R-loop instead initiates at a nick, the cost of this junction is canceled. Indeed, our data clearly show that the source of increased R-loops in nicked templates arises from R-loop initiation at, or in the immediate vicinity, of the nick (Figs. 2 and 3). Thus, unlike the addition of SSB-type proteins that increase R-loop frequencies without affecting the location of R-loops, nicks profoundly change the R-loop landscape and can transform a nearly inactive sequence into a major R-loop hotspot. Our proposal that this effect is due to the cancellation of the junction energy for nick-initiated R-loops is supported by mathematical modeling. For junction energy costs around 18 kcal/mol, the modeling data clearly show that predicted R-loop locations dramatically shift from the most favorable regions based on DNA sequence, to the position of the nick itself (*SI Appendix*).

The potential for nontemplate strand DNA nicks to act as strong initiators or R-loop formation in vitro is intriguing in the context of diseases associated with increased DNA nicks. Aicardi−Goutières syndrome (AGS), for instance, is caused by inherited mutations in nucleic acid metabolism genes (33–35) including the genes encoding for all three subunits of ribonuclease H2. RNase H2 is the primary enzyme responsible for ribonucleotide excision repair (RER), a critical pathway that removes single ribonucleotides (rNMPs) embedded in genomic DNA (36, 37). Loss of RNase H2 and AGS mutations result in accumulation of rNMPs in yeast, murine, and human genomic DNA and cause a secondary increase in DNA nicks due to the aberrant activity of DNA topoisomerase 1 at rNMPs (38). Interestingly, genome-wide mapping of RNA:DNA hybrids in AGS patient-derived fibroblasts revealed significant increases in RNA:DNA hybrid levels. Such increases, however, were relatively unique to each patient sample and were observed over DNA sequences typically devoid of favorable R-loop-forming characteristics (39). It is plausible that the sporadic accumulation of DNA nicks in RNase H2-mutated cells may cause nick-induced RNA:DNA hybrid formation. Mutations in the *ATM* gene cause Ataxia-Telangiectasia (40) and were also shown to cause increased single-strand break loads due to elevated reactive oxygen species (ROS) (41, 42). Intriguingly, ATM mutant cells also exhibit higher R-loop levels and this increased R-loop burden can be suppressed by antioxidant treatment (41), consistent with a role for ROS-induced nicks in promoting RNA:DNA hybrid accumulation. Further observations are required to determine whether the effect of DNA nicks on R-loop formation described here in vitro applies in human cells and becomes exacerbated in disease conditions. Two observations support that nicks may indeed serve as initiation sites for R-loops in cells. First, treatment of mammalian cells with the DNA topoisomerase I poison camptothecin results in a rapid increase in DNA nicks and a burst of R-loops (43, 44). Second, recent work established that strand discontinuities such as unprocessed flaps or unligated Okazaki fragments can result in a secondary accumulation of DNA:RNA hybrids in yeast (45).

In addition to increasing the local frequency of R-loop formation, nicks also appear to cause the formation of a unique type of R-loop fork structures. Direct visualization of R-loops by AFM revealed that nicks cause a 13-fold increase in such fork structures. Structure mapping, enzymatic probing, and antibody binding experiments support the notion that these structures arise from peeling and folding back of the nontemplate DNA strand as a result of the formation of an RNA:DNA hybrid. In addition, our data support that regions more prone to self-folding showed more fork structures, while regions least prone to it showed the least structures. We note that many forks also display some single-stranded character or otherwise deviate from a perfectly formed loop (*SI Appendix*, Fig. S4). Many forks also possess more than one branch (*SI Appendix*, Fig. S4*C*) indicating complex folding behavior. Furthermore, the forks we observed vary significantly in size and overall conformation, indicating they result from a dynamic folding process that can result in a variety of folded structures. Given the difficulty in imaging ssDNA by AFM, it is also possible that AFM visualization was biased toward the fork structures with the most stable pairing. Our statistics of fork frequency may therefore be an underestimate of their true rate of occurrence.

The resolution of nick-induced forked R-loops is expected to be much more demanding than that of normal R-loops. In the latter case, RNase H activity or an RNA:DNA helicase would suffice to restore B DNA once the RNA:DNA hybrid has been removed. Forked R-loops, however, would require additional factors to resolve the fork structure, including DNA helicases to unwind self-paired hairpins, an annealing activity to pair the previously peeled off nontemplate strand back to the template DNA strand, and a DNA ligase to seal the nick. The faithful resolution of forked R-loops will also require a precise choreography of enzymatic activities. If, for instance, the RNA:DNA hybrid is resolved prior to the unfolding and reattachment of the fork, a long ssDNA gap will be left on the template strand. Similarly, improper cleavage of the forked structure by a flap endonuclease may lead to the formation of a two-stranded RNA:DNA hybrid, and likely to a ssDNA gap. It is therefore likely that nick-induced forked R-loops may represent a particularly genotoxic class of R-loops that threaten genome integrity.

## Material and Methods

Engineered plasmid substrates with controlled GC content and skew were used to investigate R-loop formation through in vitro transcription (IVT) assays using T7 and T3 RNA polymerases. R-loop detection was accomplished using multiple complementary techniques including agarose gel electrophoresis, nondenaturing bisulfite conversion followed by SMRF-seq analysis, S1 nuclease digestion, and atomic force microscopy (AFM) imaging with and without S9.6 antibody treatment. We used Cas9 nickase and Nt.BspQI to introduce targeted and strand-specific nicks in plasmids, followed by IVT to investigate R-loop formation at nicks. Single-strand binding protein (SSB) experiments were conducted to examine the role of nontemplate strand stabilization in R-loop formation. Computational analysis using modified R-looper software was utilized to estimate R-loop junction energies based on a best-fit approach to our experimental data. For more information on plasmid substrates, in vitro transcription assays, nicking procedures, single-molecule R-loop footprinting, AFM imaging, and data analysis, please consult the *SI Appendix*.

## Supplementary Material

Appendix 01 (PDF)

## Data Availability

High-throughput DNA sequencing data have been deposited in Gene Expression Omnibus (GSE295014) (46).

## References

[1] J. M. Santos-Pereira, A. Aguilera, R loops: New modulators of genome dynamics and function. Nat. Rev. Genet. **16**, 583–597 (2015). 26370899 10.1038/nrg3961

[2] K. Skourti-Stathaki, N. J. Proudfoot, N. Gromak, Human senataxin resolves RNA/DNA hybrids formed at transcriptional pause sites to promote Xrn2-dependent termination. Mol. Cell. **42**, 794–805 (2011). 21700224 10.1016/j.molcel.2011.04.026 PMC3145960

[3] N. J. Proudfoot, Transcriptional termination in mammals: Stopping the RNA polymerase II juggernaut. Science **352**, aad9926 (2016). 27284201 10.1126/science.aad9926 PMC5144996

[4] L. A. Sanz , Prevalent, dynamic, and conserved R-loop structures associate with specific epigenomic signatures in mammals. Mol. Cell. **63**, 167–178 (2016). 27373332 10.1016/j.molcel.2016.05.032 PMC4955522

[5] K. Yu, F. Chedin, C.-L. Hsieh, T. E. Wilson, M. R. Lieber, R-loops at immunoglobulin class switch regions in the chromosomes of stimulated B cells. Nat. Immunol. **4**, 442–451 (2003). 12679812 10.1038/ni919

[6] C. Niehrs, B. Luke, Regulatory R-loops as facilitators of gene expression and genome stability. Nat. Rev. Mol. Cell Biol. **21**, 167–178 (2020). 32005969 10.1038/s41580-019-0206-3 PMC7116639

[7] T. García-Muse, A. Aguilera, R. Loops, From physiological to pathological roles. Cell **179**, 604–618 (2019). 31607512 10.1016/j.cell.2019.08.055

[8] S. Hamperl, K. A. Cimprich, Conflict resolution in the genome: How transcription and replication make it work. Cell **167**, 1455–1467 (2016). 27912056 10.1016/j.cell.2016.09.053 PMC5141617

[9] P. Richard, J. L. Manley, R loops and links to human disease. J. Mol. Biol. **429**, 3168–3180 (2017). 27600412 10.1016/j.jmb.2016.08.031 PMC5478472

[10] N. Sugimoto , Thermodynamic parameters to predict stability of RNA/DNA hybrid duplexes. Biochemistry **34**, 11211–11216 (1995). 7545436 10.1021/bi00035a029

[11] F. Chedin, C. J. Benham, Emerging roles for R-loop structures in the management of topological stress. J. Biol. Chem. **295**, 4684–4695 (2020). 32107311 10.1074/jbc.REV119.006364 PMC7135976

[12] R. Stolz , Interplay between DNA sequence and negative superhelicity drives R-loop structures. Proc. Natl. Acad. Sci. **116**, 6260–6269 (2019). 30850542 10.1073/pnas.1819476116 PMC6442632

[13] M. Malig, F. Chedin, “Characterization of R-Loop Structures Using Single-Molecule R-Loop Footprinting and Sequencing” in RNA-Chromatin Interactions, Methods in Molecular Biology, U. A. V. Ørom, Ed. (Springer, US, 2020), pp. 209–228. 10.1007/978-1-0716-0680-3_15 PMC7669279 32681515

[14] C.-Y. Lee , R-loop induced G-quadruplex in non-template promotes transcription by successive R-loop formation. Nat. Commun. **11**, 3392 (2020). 32636376 10.1038/s41467-020-17176-7 PMC7341879

[15] G. Miglietta, M. Russo, G. Capranico, G-quadruplex–R-loop interactions and the mechanism of anticancer G-quadruplex binders. Nucleic Acids Res. **48**, 11942–11957 (2020). 33137181 10.1093/nar/gkaa944 PMC7708042

[16] M. L. Duquette, P. Handa, J. A. Vincent, A. F. Taylor, N. Maizels, Intracellular transcription of G-rich DNAs induces formation of G-loops, novel structures containing G4 DNA. Genes Dev. **18**, 1618–1629 (2004). 15231739 10.1101/gad.1200804 PMC443523

[17] K. Zheng , Superhelicity constrains a localized and R-loop-dependent formation of G-quadruplexes at the upstream region of transcription. ACS Chem. Biol. **12**, 2609–2618 (2017). 28846373 10.1021/acschembio.7b00435

[18] O. M. Mazina , Replication protein A binds RNA and promotes R-loop formation. J. Biol. Chem. **295**, 14203–14213 (2020). 32796030 10.1074/jbc.RA120.013812 PMC7549048

[19] H. D. Nguyen , Functions of replication protein A as a sensor of R loops and a regulator of RNaseH1. Mol. Cell **65**, 832–847.e4 (2017). 28257700 10.1016/j.molcel.2017.01.029 PMC5507214

[20] D. Roy, Z. Zhang, Z. Lu, C.-L. Hsieh, M. R. Lieber, Competition between the RNA transcript and the nontemplate DNA strand during R-loop formation in vitro: A nick can serve as a strong R-loop initiation site. Mol. Cell. Biol. **30**, 146–159 (2010). 19841062 10.1128/MCB.00897-09 PMC2798282

[21] F. A. Ran , Double nicking by RNA-guided CRISPR Cas9 for enhanced genome editing specificity. Cell **154**, 1380–1389 (2013). 23992846 10.1016/j.cell.2013.08.021 PMC3856256

[22] P. Mali , CAS9 transcriptional activators for target specificity screening and paired nickases for cooperative genome engineering. Nat. Biotechnol. **31**, 833–838 (2013). 23907171 10.1038/nbt.2675 PMC3818127

[23] M. Jinek , A programmable dual-RNA-guided DNA endonuclease in adaptive bacterial immunity. Science **337**, 816–821 (2012). 22745249 10.1126/science.1225829 PMC6286148

[24] P. A. Ginno, P. L. Lott, H. C. Christensen, I. Korf, F. Chédin, R-loop formation is a distinctive characteristic of unmethylated human CpG island promoters. Mol. Cell **45**, 814–825 (2012). 22387027 10.1016/j.molcel.2012.01.017 PMC3319272

[25] D. D. Phillips , Antibody recognition of DNA-RNA hybrids.. J. Mol. Recognit. **26**, 376–381 (2013). 23784994 10.1002/jmr.2284 PMC4061737

[26] W. R. Bauer, C. J. Benham, The free energy, enthalpy and entropy of native and of partially denatured closed circular DNA. J. Mol. Biol. **234**, 1184–1196 (1993). 8263920 10.1006/jmbi.1993.1669

[27] Y. Carrasco-Salas , The extruded non-template strand determines the architecture of R-loops. Nucleic Acids Res. **47**, 6783–6795 (2019). 31066439 10.1093/nar/gkz341 PMC6648340

[28] S. Broccoli , Effects of RNA polymerase modifications on transcription-induced negative supercoiling and associated R-loop formation: RNA polymerase in transcription-induced supercoiling. Mol. Microbiol. **52**, 1769–1779 (2004). 15186424 10.1111/j.1365-2958.2004.04092.x

[29] N. J. Bonde, A. G. Kozlov, M. M. Cox, T. M. Lohman, J. L. Keck, Molecular insights into the prototypical single-stranded DNA-binding protein from *E. coli*. Crit. Rev. Biochem. Mol. Biol. **59**, 99–127 (2024). 38770626 10.1080/10409238.2024.2330372 PMC11209772

[30] S. Saxena, L. Zou, Hallmarks of DNA replication stress. Mol. Cell **82**, 2298–2314 (2022). 35714587 10.1016/j.molcel.2022.05.004 PMC9219557

[31] A. Maréchal, L. Zou, RPA-coated single-stranded DNA as a platform for post-translational modifications in the DNA damage response. Cell Res. **25**, 9–23 (2015). 25403473 10.1038/cr.2014.147 PMC4650586

[32] R. E. Brown , The RNA export and RNA decay complexes THO and TRAMP prevent transcription-replication conflicts, DNA breaks, and CAG repeat contractions. PLoS Biol. **20**, e3001940 (2022). 36574440 10.1371/journal.pbio.3001940 PMC9829180

[33] J. Aicardi, F. Goutières, A progressive familial encephalopathy in infancy with calcifications of the basal ganglia and chronic cerebrospinal fluid lymphocytosis. Ann. Neurol. **15**, 49–54 (1984). 6712192 10.1002/ana.410150109

[34] Y. J. Crow , Characterization of human disease phenotypes associated with mutations in TREX1, RNASEH2A, RNASEH2B, RNASEH2C, SAMHD1, ADAR, and IFIH1. Am. J. Med. Genet. A. **167**, 296–312 (2015). 10.1002/ajmg.a.36887 PMC4382202 25604658

[35] C. Uggenti , Cgas-mediated induction of type I interferon due to inborn errors of histone pre-mRNA processing. Nat. Genet. **52**, 1364–1372 (2020). 33230297 10.1038/s41588-020-00737-3

[36] S. M. Cerritelli, R. J. Crouch, Ribonuclease h: The enzymes in eukaryotes: Ribonucleases h of eukaryotes. FEBS J. **276**, 1494–1505 (2009). 19228196 10.1111/j.1742-4658.2009.06908.x PMC2746905

[37] M. A. M. Reijns, A. P. Jackson, Ribonuclease H2 in health and disease. Biochem. Soc. Trans. **42**, 717–725 (2014). 25109948 10.1042/BST20140079

[38] N. Kim , Mutagenic processing of ribonucleotides in DNA by yeast topoisomerase I. Science **332**, 1561–1564 (2011). 21700875 10.1126/science.1205016 PMC3380281

[39] Y. W. Lim, L. A. Sanz, X. Xu, S. R. Hartono, F. Chédin, Genome-wide DNA hypomethylation and RNA:DNA hybrid accumulation in Aicardi-Goutières syndrome. eLife **4**, e08007 (2015). 26182405 10.7554/eLife.08007 PMC4528086

[40] C. Rothblum-Oviatt , Ataxia telangiectasia: A review. Orphanet J. Rare Dis. **11**, 159 (2016). 27884168 10.1186/s13023-016-0543-7 PMC5123280

[41] P. R. Woolley , Regulation of transcription patterns, poly(ADP-ribose), and RNA-DNA hybrids by the ATM protein kinase. Cell Rep. **43**, 113896 (2024). 38442018 10.1016/j.celrep.2024.113896 PMC11022685

[42] T. T. Paull, P. R. Woolley, A-T neurodegeneration and DNA damage-induced transcriptional stress. DNA Repair. **135**, 103647 (2024). 38377644 10.1016/j.dnarep.2024.103647 PMC11707827

[43] J. Marinello, G. Chillemi, S. Bueno, S. G. Manzo, G. Capranico, Antisense transcripts enhanced by camptothecin at divergent CpG-island promoters associated with bursts of topoisomerase I-DNA cleavage complex and R-loop formation. Nucleic Acids Res. **41**, 10110–10123 (2013). 23999093 10.1093/nar/gkt778 PMC3905886

[44] R. C. Duardo , Human DNA topoisomerase I poisoning causes R loop–mediated genome instability attenuated by transcription factor IIS. Sci. Adv. **10**, eadm8196 (2024). 38787953 10.1126/sciadv.adm8196 PMC11122683

[45] R. M. Mangione , DNA lesions can frequently precede DNA:RNA hybrid accumulation. Nat. Commun. **16**, 2401 (2025). 40064914 10.1038/s41467-025-57588-x PMC11893903

[46] E. Holleman , Data from “Protein-mediated stabilization and nicking of the non-template DNA strand dramatically affect R-loop formation in vitro.” Gene Expression Omnibus. https://www.ncbi.nlm.nih.gov/geo/query/acc.cgi?acc=GSE295014. Deposited 3 September 2025. 10.1073/pnas.2509309122 PMC12478114 40966285

