# Supplementary material for: Protein-mediated stabilization and nicking of the nontemplate DNA strand dramatically affect R-loop formation in vitro
Source: Proc Natl Acad Sci U S A. 2025 Sep 18;122(38):e2509309122. doi: 10.1073/pnas.2509309122 (PMC12478114; doi:10.1073/pnas.2509309122)
Supplement: Supplementary file 1 — Appendix 01 (PDF) [file pnas.2509309122.sapp.pdf]

Supporting Information Appendix for:

Protein-mediated stabilization and nicking of the non-template DNA strand dramatically affect R-loop formation *in vitro*

Ethan Holleman[1], Thomas E. Catley[2], Tadas Sereiva[1], Stella Hartono[1], Alice L.B. Pyne[2], and Frédéric Chédin[1,*]

CORRESPONDENCE

May be addressed to Frédéric Chédin at flchedin@ucdavis.edu

This PDF file includes:

- **Supporting text**
- **Figures S1 to S6**
- **SI References**

Supporting Information text:

Estimation of the energetic cost of the R-loop junctional energy

Previously, R-loop modeling using R-looper (1) assumed that the junction energy parameter (*a*) was 10 kcal/mol, following earlier experimental measurements for B/Z transitions and local strand separation (2–4). The junction energy for R-loops has not been experimentally determined but could be higher than simple strand separation given that R-loops must accommodate the RNA strand of the RNA:DNA hybrid inside a strand-separated bubble. To empirically model (*a*), we modified R-looper such that no junction energy would be applied specifically for R-loops initiating at nicks. We assumed that negative superhelicity was -7% for these calculations, consistent with measurement of transcription-induced dynamic supercoiling (5). The junction energy itself was varied systematically and R-looper was used to generate simulated R-loop footprints. Focusing on the distal position 3 for which a nick caused a two-order of magnitude increase in R-loop frequencies, we observed that for a default (*a*) value of 10 kcal/mol, most R-loops are predicted to form around the central, energetically favorable region, regardless of whether junction energy relief was applied at the nick (**Fig S6A**). As the value of (a) is increased, however, the cost of initiating R-loops there is raised. By contrast, initiation at the nick, where the increasing junction energy cost does not apply, is becoming progressively more likely, outweighing the penalty associated with initiating in a poor R-loop forming DNA sequence. At an (a) value of 17.9 kcal/mol, we observed the best fit for simulated vs. observed R-loop footprints (**Fig S6B**), as measured by the Precision value. Under that condition, most R-loops are now predicted to form at the nick, simulating a dramatic shift of R-loop positions. This data therefore suggests that the critical junction energy value (a) is significantly higher than previously thought. We note, however, that the (a) value estimate is likely to vary with the local DNA sequence and changes considerably with the level of available negative DNA superhelicity. As the negative supercoiling is increased, the value of (a) needed to shift the distribution of R-loops to the nick sites is also strongly increased (**Fig S6C**). This is consistent with the notion that to observe shifting to the nick, one must increasingly penalize the formation of R-loops over the most favorable regions based on DNA sequence and topology. Regardless, our data and modeling suggest that the value of (a) has been previously under-estimated and that nicks derive their ability to focus R-loop initiation from the relief of this otherwise severely limiting parameter.

Materials and methods

Plasmid substrates.

The VR-10 and VR-20 plasmids were cloned by digesting the pFC9 SNRPN containing plasmid (1, 6) with SacI-HF® (NEB, R3156S) and EcoRI-HF® (NEB, R3101S) according to the manufacturer's online protocol. The large fragment resulting from the digest was then separated via agarose gel electrophoresis and subsequently extracted. Synthetic DNA sequences, or variable regions (VRs) with corresponding pFC9 homology sites of 250 bp in length were then inserted via Gibson assembly (7) into the pFC9 based vector. The VR sequences were designed to have specific R-loop forming properties, namely controlled GC and content. VR-10 was designed to have an overall GC skew and content of 0.2 and 0.5 while VR-20 was designed with 0.4 and 0.6 respectively. All guanines within the VR-20 sequence were clustered into triplets and distributed at a regular interval through the sequence while the guanines in VR-10 were allowed to distribute randomly. The VR-10 and 20 plasmid sequences were confirmed via Sanger sequencing.

Plasmid isolation

Plasmids were transformed into *E. coli* DH5α chemically competent cells and single colonies were grown on LB ampicillin agar plates. Single colonies were selected and grown in 300 ml LB culture with 100 µg / ml of ampicillin in 1 L flasks at either 30° or 37°C overnight with shaking at 200-250 rpm. Negatively supercoiled plasmid substrates were purified by alkaline lysis protocol followed by column cleanup (Qiagen) and eluted into either TE or EB buffer.

In vitro transcription reactions

In vitro transcription assays were performed as described in Ginno *et al.*, (2012) (6) with 20 ng / µl DNA and either T7 (NEB M0251S) or T3 (NEB M0378S) RNA polymerases (2.5 U / µl) in the buffer provided by the polymerase manufacturer supplemented with rNTP mix solution (NEB, N0466S) added to a final concentration of 0.5 mM and DTT (NEB, B1222A) to a final concentration of 10 mM. Reactions were incubated at 37°C for 20 minutes and then were terminated by the addition of EDTA to a final concentration of 35 mM or by physical separation of the promoter from the transcribed region by restriction digest. For direct visualization, the reaction products were treated with RNase A (33 ng/ml) for 30-45 minutes at 37°C to degrade

excess free RNA. To confirm presence of R-loops, a portion of the transcribed sample was treated with RNase H (NEB M0297S, 40 ng DNA/1U) for 30 minutes at 37° C to degrade RNA:DNA hybrids. The products were then separated by agarose gel electrophoresis using 0.8% 1x TBE gel at a constant 60 volts for 2 hours. The gels were then post stained with ethidium bromide and imaged with a BioRad GelDoc EZ imager.

Design and synthesis of single guide RNAs

Specific Cas9 single guide RNAs (sgRNAs) target sites were selected using Benchling online software. Oligos with the selected target sites were ordered according to the NEB EnGen® sgRNA Synthesis Kit, *S. pyogenes* (E3322V) kit protocol. sgRNAs were then synthesized according to NEB protocols and purified via phenol-chloroform ethanol precipitation. The concentration of purified sgRNAs was determined by measuring absorbance with a NanoDrop microvolume spectrophotometer.

Cas9 nickase digestion and linearization of supercoiled plasmids

Cas9 nickase (NEB M0650T) and sgRNA solutions were prepared according to NEB EnGen® Spy Cas9 Nickase digestion protocol with 300 nM sgRNA and 30 nM Cas9 nickase. The reaction mixture was then allowed to incubate at room temperature for 10 minutes. Supercoiled plasmid DNA was then added to the reaction mixture at a final concentration of 3 nM. The reaction was then incubated at 37°C for 15 minutes after which it was placed on ice and digested with Proteinase K for 30 minutes. The reaction products were then phenol-chloroform ethanol precipitated and resuspended in a 10 mM Tris HCl buffer. Nicking efficiency was assayed via agarose gel electrophoresis by running the nicked product against an untreated supercoiled control. Reactions that showed greater than or equal to 95% nicked product as evidenced by the loss of mobility after conversion from a supercoiled to open-circle state were considered fully nicked and utilized for downstream assays. Samples were then digested with BsaI-HF®v2 (NEB R3733S) according to the manufacturer's instructions. The reaction products were digested for 30 minutes at room temperature using Proteinase K (Roche, RPROTK-RO) and then were then purified via phenol-chloroform extraction followed by ethanol precipitation. The position of nicks and nicking efficiency was verified by Sanger sequencing using primers selected to hybridize to the nicked strand. A nick was confirmed through this method if the sequencing trace was terminated early at the expected site of the nick but successful in a

complementary untreated control reaction. Sequencing was performed at the UC Davis Sanger sequencing core facility.

Cloning of pFC9ΔNt.BspQI series plasmids

pFC9 plasmid was digested with XbaI (NEB R0145S) and SapI-HF® (NEB R3156S) according to the manufacturer's instructions to remove an existing Nt.BspQI recognition site. The large fragment resulting from this digest was then isolated via an agarose gel extraction. The plasmid ends were blunted using DNA Polymerase I, Large (Klenow) Fragment (NEB M0210) according to the manufacturer's blunting protocol. The reaction products were isolated by phenol-chloroform extraction followed by ethanol precipitation and then ligated using T4 DNA ligase overnight at 16°C. The resulting ligated products were then transformed into DH5α competent cells. Single colonies were isolated, grown overnight, and plasmid was extracted via alkaline lysis followed by lithium chloride extraction and ethanol precipitation. The entire construct was then amplified with three independent sets of PCR primers using Q5 high fidelity DNA polymerase (NEB M0492S), each resulting in an insertion of an approximately 3.5 kb product with a Nt.BspQI site inserted at the forward primer binding site. The PCR products were then purified as previously described by agarose gel extraction and ligated using T4 ligase overnight at 16°C. The ligated products were then transformed into DH5α cells, grown up from single colonies, and plasmid DNA was isolated as previously described. The location and sequence of the Nt.BspQI insertions were verified via Sanger sequencing. Supercoiled plasmids were subsequently digested with Nt.BspQI (NEB R0644S) according to the manufacturer's online protocol. Reaction products were purified using DNA binding spin columns and complete nicking of the substrate was verified in the same manner as Cas9 nickase-treated samples.

Estimation of R-loop junction free energy

The R-looper (https://github.com/chedinlab/rlooper_sim) source code was modified to allow for a zero junction energy value (a parameter) to be applied at a specific base pair range within a provided DNA sequence. Junction energy was then set to zero kcal/mol in a 20 bp region surrounding the location of Cas9 / Nt.BspQI induced nicks. A non-zero junction energy value was applied normally to all other base pair positions in the sequence. The modified R-looper was then run with increasing junction energy values for all other regions outside of the nick

region. The position of predicted R-loops was then compared to experimental data to determine the junction energy which provided the best fit to the experimental data.

In vitro transcription with single strand binding protein

IVT reaction mixtures was prepared as previously described in *In vitro transcription reactions.* Co-SSB samples were supplemented with purified *E.coli* single-strand binding protein (8) (SSB; a kind gift from Dr. Stephen C. Kowalczykowski) to a final concentration of 2.5 μM before the addition of the appropriate polymerase. After termination of the transcription reaction, SSB was added to a final concentration of 2.5 μM to Post-SSB samples and allowed to incubate at 37° C for 20 minutes. Both Co and Post SSB samples were then digested with Proteinase K at 37° C for 30 minutes and then purified via phenol-chloroform extraction followed by ethanol precipitation. After the deproteinization and precipitation steps the samples were then bisulfite treated as described in *Non-denaturing bisulfite conversion.*

Non-denaturing bisulfite conversion

Non-denaturing bisulfite conversion of plasmid substrates was performed as previously described (9) using the EZ DNA Methylation-Lightning Kit (Zymogen D5030). Nicked samples were then treated overnight at 16°C with T4 DNA ligase (NEB M0202S) according to the manufacturer's provided online protocol. This repair step ensures that the nicked strand can be PCR amplified, and R-loop signal can be subsequently measured.

SMRF-seq sample barcoding, PCR amplification, sequencing and analysis

Samples were amplified with a combination of forward and reverse primers that each incorporated a unique barcode. The combination of the forward and reverse primer barcodes uniquely identifies each sample within its specific flowcell. Primer binding sequences were designed in Primer3 (10) software and barcodes were generated using a custom Python script. Samples were amplified using Q5U® Hot Start High-Fidelity DNA Polymerase (NEB M0515S) for 25-30 cycles according to the manufacturer's online protocol. 1-3 μl of bisulfite converted substrate was used for each reaction. In the case that the yield of one reaction was not sufficient, additional reactions using the original bisulfite converted sample were performed and then pooled. The target amplicon size was then purified via agarose gel extraction using

BioBasic gel extraction kit (BS654). Sample concentration was quantified via agarose gel electrophoresis against 1kb linear DNA ladder (Thermo Fisher SM0311). SMRT cell PacBio libraries were constructed by the UC Davis DNA Technologies core according to manufacturer's instructions and sequenced on a PacBio Sequel II instrument. Additional sequencing was performed at the California Institute for Quantitative Biosciences at UC Berkeley (QB3-Berkeley) on a PacBio Revio instrument. Fastq files were processed, and R-loop footprints were called using the FootLoop program (https://github.com/srhartono/footLoop). R-loops were called using minimum parameters of 50 base pair minimum peak length (-t), at least 20 cytosines present in the putative R-loop (-w), and a minimum cytosine conversion rate of 35% (-t).

Assay for T7 initiation at nick sites

Supercoiled pFC9 was either nicked with Nt.BspQI or left untreated and digested with either a single digest of BamHI (NEB) or BamHI-HF and SacI-HF. The BamHI in this plasmid is located 5 bp upstream of the T7 promoter sequence while the SacI site is located 6 bp downstream. Therefore, digestion with only BamHI leaves the T7 promoter connected to the downstream R-loop forming SNRPN region while linearizing while digestion with both enzymes removes the T7 promoter from the larger plasmid as a ~42 bp fragment. After digestion the large fragment of all digests were purified via agarose gel extraction and eluted into EB buffer. 400 ng of each sample was then used as input into an IVT. Each of the four samples were split into transcribed and un-transcribed aliquots and the IVT reaction using T7 RNAP was carried out as previously described. After the reaction the products were run on a 0.8% 1x TBE agarose gel for 2 hours at 60V. The gel was then post-stained with EtBr for 20 minutes and imaged.

In vitro transcription followed by S1 nuclease digestion

In vitro transcription reactions were performed as described. Samples were purified after termination of the transcription reaction via phenol-chloroform extraction followed EtOH precipitation. Samples were resuspended in 1x S1 nuclease buffer (Takara Bio 2410B) supplemented with $MgCl_2$ to a final concentration of 6 mM to allow for simultaneous digestion with S1 nuclease (Takara Bio 2410B) and RNase H. RNase A was added to all samples at a final concentration of 5 ng/ µg. S1 nuclease was added to digested samples to a final concentration of 2 units / 100 ng of DNA and 3 units RNase H were added to digested samples. The reactions were kept on ice during preparation to avoid excessive digestion by S1 nuclease.

All samples were brought to a final volume of 20 µl using PCR grade water and then incubated at 37°C for 30 minutes. After the incubation samples were removed and placed on ice and run on 1x TBE 0.8% agarose gel for two hours at 60V. The gel was then post-stained in EtBr for approximately 30 minutes, destained in DI water for 10 minutes, and then imaged using a BioRad GelDoc imager.

DNA secondary structure predictions

The complete sequence of pFC9 with the origin set at the PsiI recognition site to reflect sample digestion for AFM imaging was input into the RNAfold webserver (11) (http://rna.tbi.univie.ac.at/cgi-bin/RNAWebSuite/RNAfold.cgi) using the DNA parameters setting (12) and default parameters otherwise.

AFM sample preparation

For plasmid only samples, 10-15 ng of DNA was immobilized on a freshly cleaved mica disk in 20 µl of immobilization buffer (25 mM MgCl2, 10 mM Tris-HCl, pH 7.4) for 5 min. The mica was then washed 4 times with 20 µl imaging buffer (3 mM NiCl2, 20 mM HEPES, pH 7.4), before and a further 20 µl was added for imaging. For the S9.6 antibody binding experiment, 10 ng of DNA was pre-incubated with 1 ng of Anti-DNA-RNA Hybrid Antibody, clone S9.6 (Sigma Aldrich), and 7 µl of 1X DRIP buffer for 20 mins at room temperature. This reaction was then immobilized as previously in immobilization buffer (25 mM MgCl2, 10 mM Tris-HCl, pH 7.4) for 5 min, before washing and imaging in imaging buffer (3 mM NiCl2, 20 mM HEPES, pH 7.4).

AFM imaging

All AFM measurements were performed in liquid following a previously published protocol (13). All experiments were carried out in PeakForce Tapping imaging mode on either a Multimode 8 (Bruker) or FastScan Dimension XR AFM system (Bruker), using either PeakForce HRS-F-B (Bruker) or FastScan D (Bruker) probes. The PeakForce amplitude was set to 6-10 nm, the PeakForce Tapping frequency to 2-8 kHz and the PeakForce setpoints in the range: 7-15 mV, corresponding to peak forces of <70 pN. Various scan sizes were taken, maintaining a resolution of < 2 nm/px, at line rates of ~1-5 Hz.

AFM image analysis

The freely available, open-source software TopoStats (14) was used to process the AFM data and analyze the DNA molecules (https://github.com/AFM-SPM/TopoStats). Briefly, the software loaded raw AFM images, carried out flattening, both line-by-line and plane flattening. Individual molecules were masked based on a height threshold to separate them from the background. A second flattening was carried out which excluded the grain, improving the flattening of the image. The height distribution of the flattened image was then shifted vertically to set the background to zero by calculating the mean of the non-grain containing data and subtracting that value from the image. Finally, a 1.1 px Gaussian filter was applied to reduce any high-gain noise. For classification and quantification of the R-loop features, the image analysis software FIJI was used to import the processed image files and manually trace the DNA strands. The features were classified as either “Blobs”, “Loops” or “Forks” depending on their morphology. The total contour length of the plasmids was measured as well as the distance to the feature and the length of the feature, if present.

Supplementary figures and figure legends.

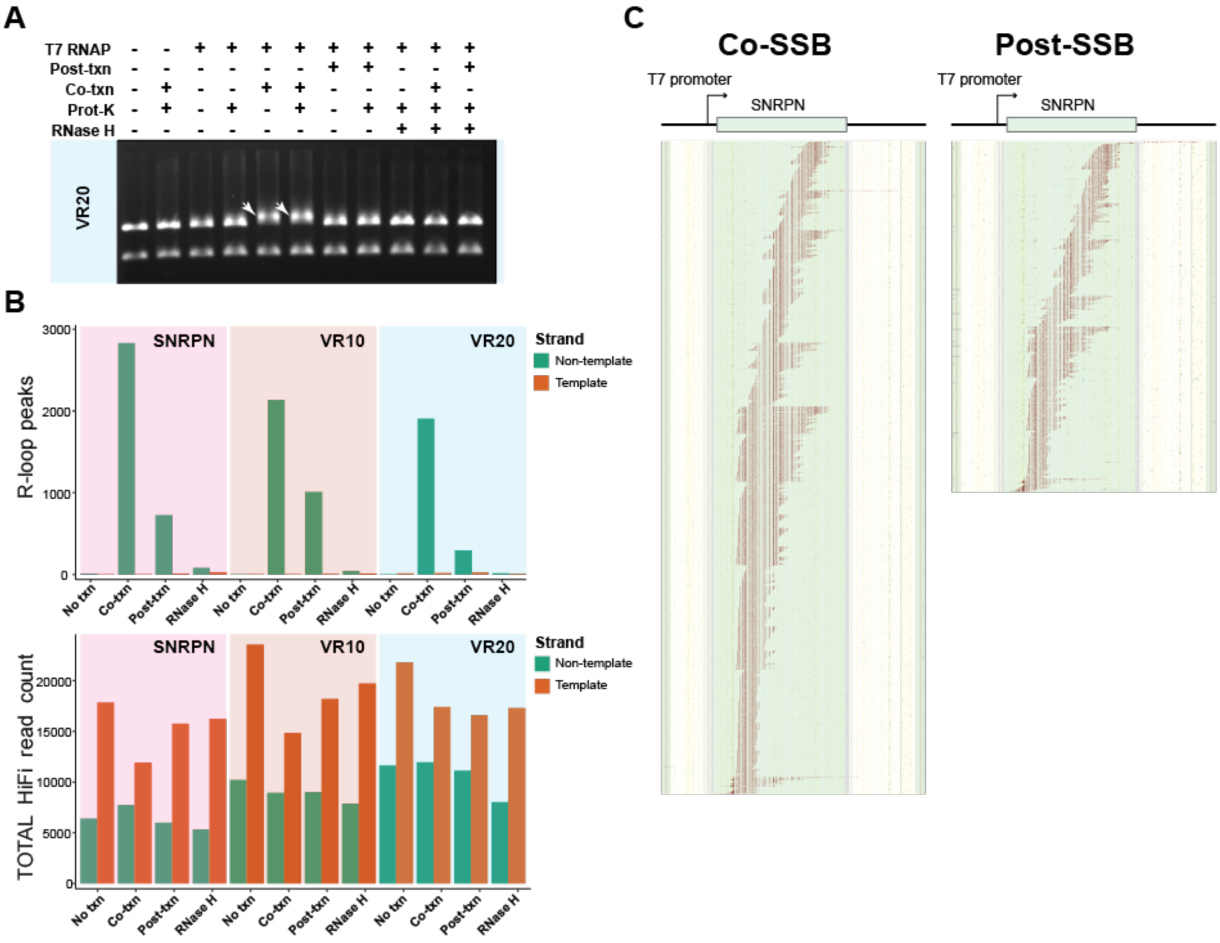

Figure S1. A: Agarose gel electrophoresis of the products of *in vitro* transcription reaction on the linearized VR20 plasmid under co and post SSB conditions. Upwards shifting indicating R-loop formation are indicated on lanes 5 and 6. Upward shifting is not reduced by Proteinase K treatment indicating this is not a result of a reduction of mobility due to SSB binding. This shifting is also sensitive to RNase H treatment (lanes 10 and 11). **B:** Barplot showing the number of measured R-loop peaks for all plasmids and SSB treatments utilized. **C:** Barplot of total number of reads for all plasmids and SSB treatments. **C:** R-loop footprints from *in vitro* transcription reactions of SNRPN containing plasmid under both co and post SSB conditions.

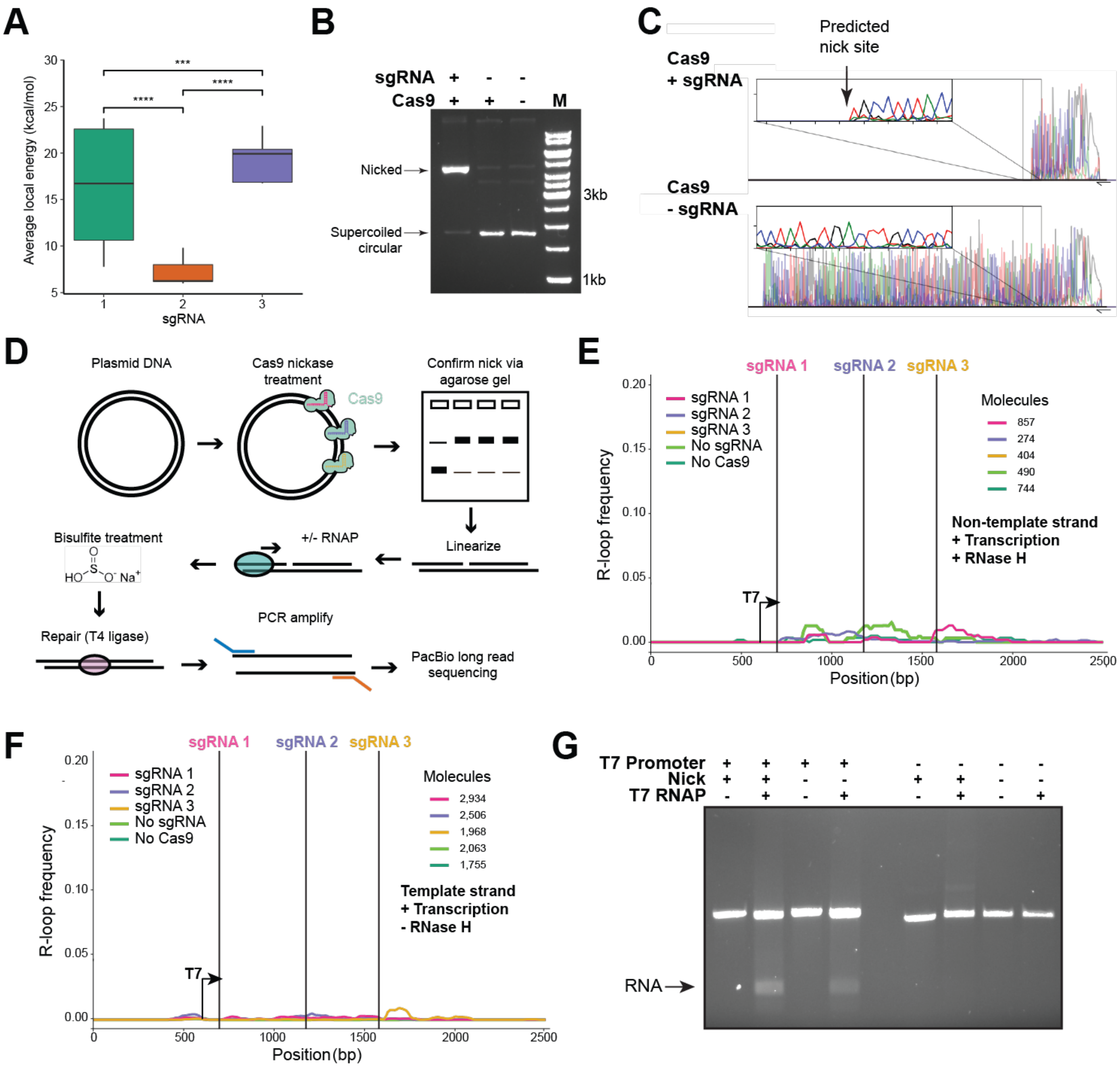

Figure S2. A: Boxplot showing local average energy in kcal / mol calculated using the R-looper model for the sequences within 50 bp of Cas9-induced nicks in the *SNRPN* region. **B:** Electrophoretic mobility shift assay comparing Cas9 and sgRNA-treated and untreated supercoiled DNA. **C**: Read traces from Sanger sequencing of a Cas9 and sgRNA-treated and untreated samples. The location of the predicted nick is highlighted. **D:** Diagram of the adapted SMRF-seq procedures for nicked substrates. See Sup Methods for additional details. **E**: R-loop frequency plot calculated from SMRF-seq data from the template strand of Cas9-nicked and unnicked plasmids. In this transcriptional context R-loop formation is expected on the non-

template strand. **F**: R-loop frequency plot calculated from SMRF-seq data from transcribed and RNase H-treated Cas9-nicked and unnicked *SNRPN* containing plasmids on the R-loop-prone non-template strand. **G:** Agarose gel electrophoresis of the products of *in vitro* transcription reaction on linearized Cas9-nicked and unnicked plasmids containing, or not, the T7 promoter. RNA produced by transcription by T7 RNA polymerase can be seen as a smear at the bottom of the gel. Only plasmids containing the T7 promoter produced RNA regardless of their nicking status.

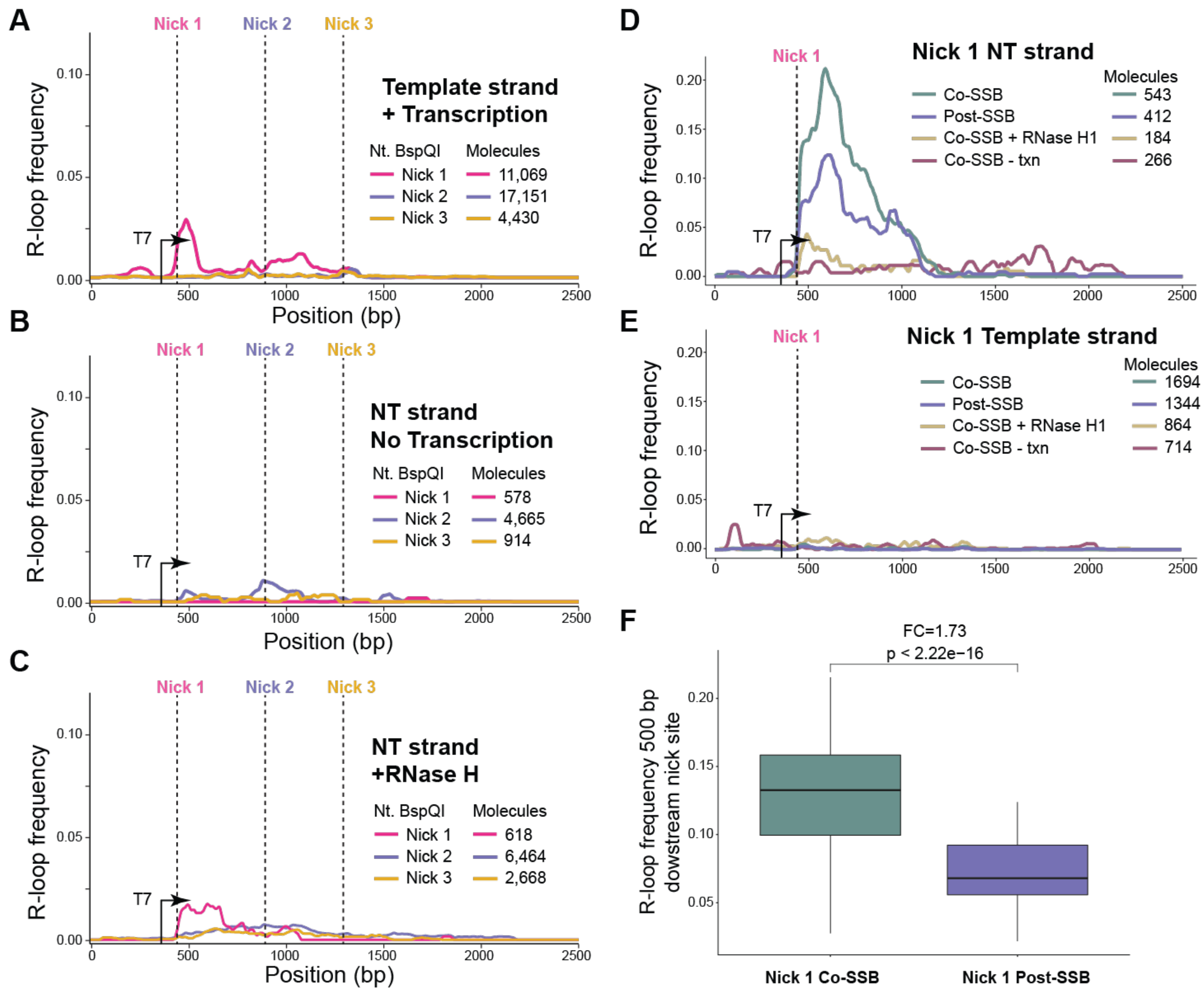

Figure S3. A: R-loop frequency plot from transcribed and Nt.BspQI-nicked plasmids calculated from SMRF-seq data on the template strand. **B:** R-loop frequency plot calculated from SMRF-seq data from untranscribed and Nt.BspQI-nicked substrates on the non-template strand. **C:** R-loop frequency plot calculated from SMRF-seq data from transcribed and RNase H-treated Nt.BspQI-nicked substrates on the non-template strand. **D:** R-loop frequency plot calculated from SMRF-seq data of the non-template strand of nicked and co-txn SSB treated samples. The dotted line indicates the position of the nick 1 site. **E:** R-loop frequency plot from SMRF-seq data of the template strand of nicked and co-txn SSB treated samples. **F:** Boxplot comparing R-loop frequency 500 bp downstream of the nick 1 site in nicked co-txn SSB or post-txn SSB treated samples. Fold change and p-value calculated from student's t-test are indicated.

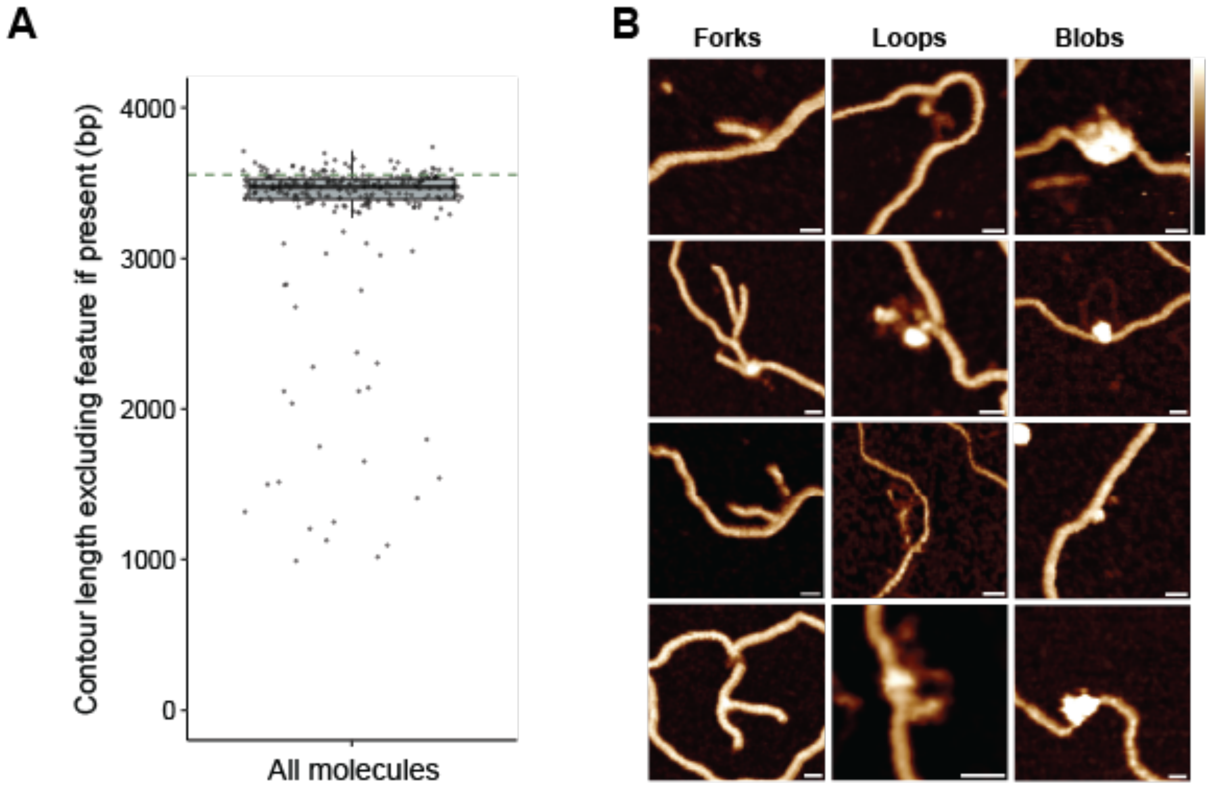

Figure S4. A: Boxplot showing distribution of molecule contour lengths measured via AFM in base pairs. The horizontal dashed green line shows the expected molecule size. **B:** Additional AFM images of the three classes of observed features; forks, loops and blobs. Scale bars indicate 10 nm. Height scale = -1 to 3 nm.

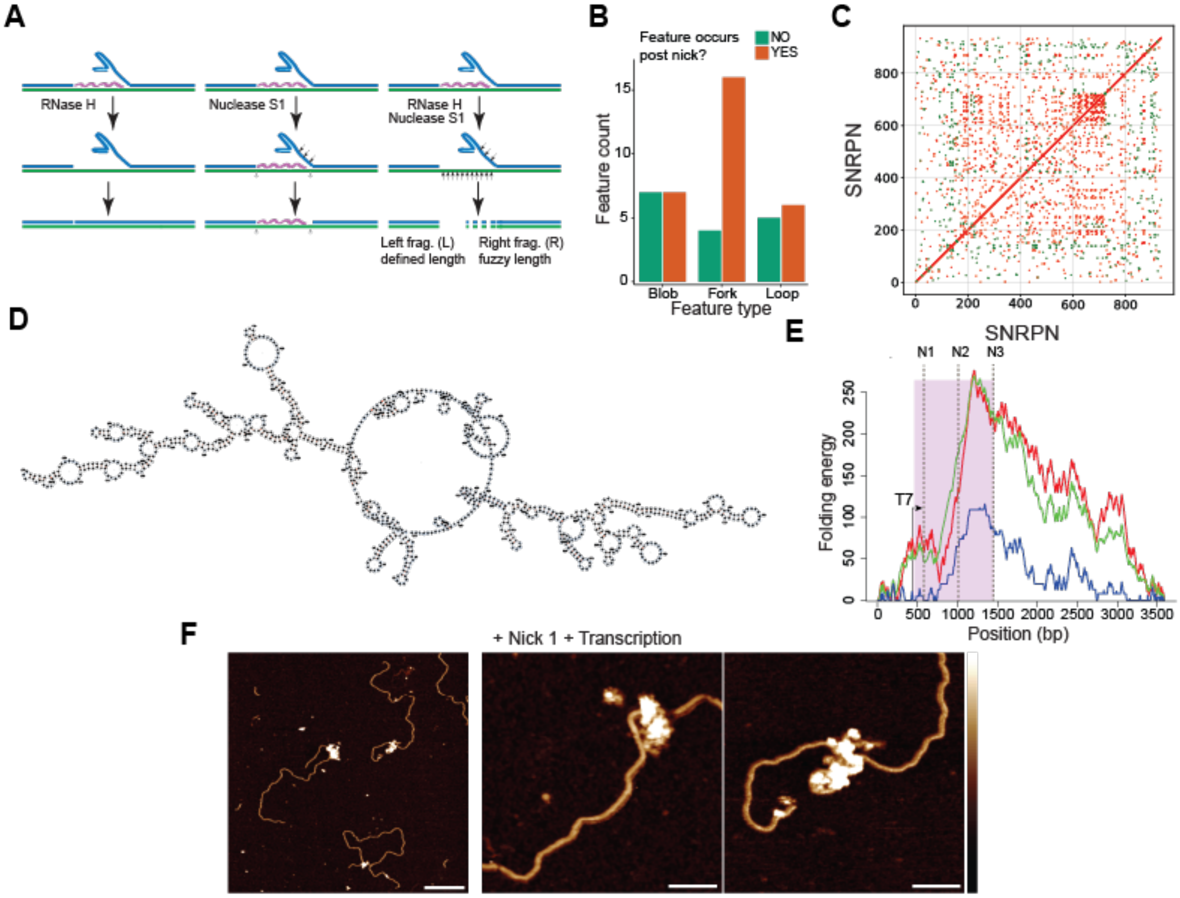

Figure S5. A: Model outcome for the digestion of a forked R-loop with RNase H, S1 nuclease, and both. Cleavage sites are indicated by small arrows. **B:** Barplot showing the number of features, by feature type, that occur either before or after the site of a nick. **C:** Dot plot of SNRPN sequence showing direct repeats (red) and inverted repeats (green); kmer size = 5, exact matches only. **D:** Free-energy minimizing secondary structure prediction for the *SNRPN* sequence. **E:** DNA secondary structure favorability prediction over the *SNRPN* containing plasmid. The *SNRPN* region is highlighted in purple and the site of each of the three nicks are labeled and shown with dashed vertical lines. The location and direction of the T7 promoter is also shown. **F:** Representative image of S9.6 antibody binding in linearized and transcribed samples with a nick present at location 1. Left-most image scale bar = 200 nm, middle and right-most images scale bars = 20 nm. Height scale = -1 to 3 nm. The right two images are zoomed-in views of the structures present in the left image.

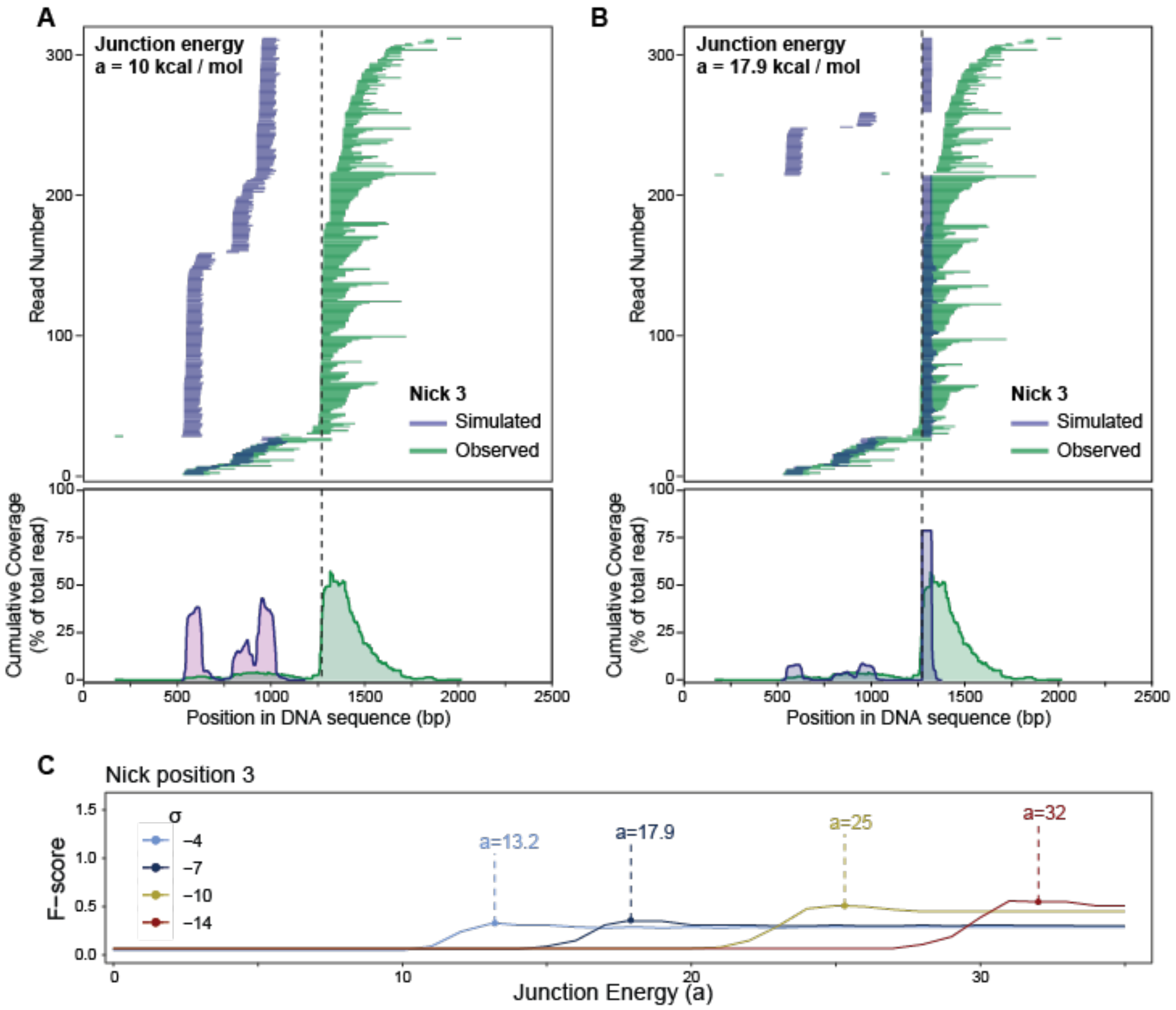

Figure S6: A: Observed (via SMRF-seq) R-loop locations from transcription of *SNRPN* containing plasmid nicked using Cas9 and sgRNA 3 vs. simulated R-loop locations from R-looper using the default junctional energy (a) value of 10 kcal / mol. The location of the nick is shown with a vertical dashed line. The top plot shows individual R-loop footprints while the bottom displays R-loop coverage. **B:** Identical to **A** but a junctional energy of 17.9 kcal / mol was utilized in the R-looper simulation. **C:** F-score plotted as a function of junction energy (a) for increasing levels of negative supercoiling (σ). The a value at the point with the highest f-score for each value of σ is shown.

SI references

1. R. Stolz, *et al.*, Interplay between DNA sequence and negative superhelicity drives R-loop structures. *Proc. Natl. Acad. Sci.* **116**, 6260–6269 (2019).
2. C. J. Benham, Energetics of superhelicity and of B-Z transitions in superhelical DNA. *Cell Biophys.* **10**, 193–204 (1987).
3. W. R. Bauer, C. J. Benham, The free energy, enthalpy and entropy of native and of partially denatured closed circular DNA. *J. Mol. Biol.* **234**, 1184–1196 (1993).
4. D. Zhabinskaya, C. J. Benham, Theoretical Analysis of the Stress Induced B-Z Transition in Superhelical DNA. *PLOS Comput. Biol.* **7**, e1001051 (2011).
5. F. Kouzine, *et al.*, Transcription-dependent dynamic supercoiling is a short-range genomic force. *Nat. Struct. Mol. Biol.* **20**, 396–403 (2013).
6. P. A. Ginno, P. L. Lott, H. C. Christensen, I. Korf, F. Chédin, R-Loop Formation Is a Distinctive Characteristic of Unmethylated Human CpG Island Promoters. *Mol. Cell* **45**, 814–825 (2012).
7. D. G. Gibson, *et al.*, Enzymatic assembly of DNA molecules up to several hundred kilobases. *Nat. Methods* **6**, 343–345 (2009).
8. M. S. Wold, J. B. Mallory, J. D. Roberts, J. H. Lebowitz, R. Mcmacken, Initiation of bacteriophage A DNA replication in vitro with purified A replication proteins.
9. M. Malig, F. Chedin, “Characterization of R-Loop Structures Using Single-Molecule R-Loop Footprinting and Sequencing” in *RNA-Chromatin Interactions*, Methods in Molecular Biology., U. A. V. Ørom, Ed. (Springer US, 2020), pp. 209–228.
10. A. Untergasser, *et al.*, Primer3—new capabilities and interfaces. *Nucleic Acids Res.* **40**, e115 (2012).
11. A. R. Gruber, R. Lorenz, S. H. Bernhart, R. Neubock, I. L. Hofacker, The Vienna RNA Websuite. *Nucleic Acids Res.* **36**, W70–W74 (2008).
12. D. H. Mathews, Using an RNA secondary structure partition function to determine confidence in base pairs predicted by free energy minimization. *RNA* **10**, 1178–1190 (2004).

13. P. J. Haynes, K. H. S. Main, B. Akpinar, A. L. B. Pyne, “Atomic Force Microscopy of DNA and DNA-Protein Interactions” in *Chromosome Architecture: Methods and Protocols*, M. C. Leake, Ed. (Springer US, 2022), pp. 43–62.
14. J. G. Beton, *et al.*, TopoStats – A program for automated tracing of biomolecules from AFM images. *Methods* **193**, 68–79 (2021).
15. J. Schindelin, *et al.*, Fiji: an open-source platform for biological-image analysis. *Nat. Methods* **9**, 676–682 (2012).
